# Genome-wide identification and functional analyses of microRNA signatures associated with cancer pain

**DOI:** 10.1002/emmm.201302797

**Published:** 2013-10-18

**Authors:** Kiran Kumar Bali, Deepitha Selvaraj, Venkata P Satagopam, Jianning Lu, Reinhard Schneider, Rohini Kuner

**Affiliations:** 1 Medical Faculty Heidelberg, Institute for Pharmacology, Heidelberg UniversityHeidelberg, Germany; 2 Molecular Medicine Partnership Unit with European Molecular Biology LaboratoryHeidelberg, Germany; 3 Luxembourg Centre for Systems Biomedicine (LCSB), University of Luxembourg, Campus Belval, House of BiomedicineEsch-sur-Alzette, Luxembourg; 4 European Molecular Biology LaboratoryHeidelberg, Germany

**Keywords:** bone metastatic pain, Clcn3, gene regulation, miRNA inhibitors, miRNA mimics

## Abstract

Cancer pain remains a major challenge and there is an urgent demand for the development of specific mechanism-based therapies. Various diseases are associated with unique signatures of expression of microRNAs (miRNAs), which reveal deep insights into disease pathology. Using a comprehensive approach combining genome-wide miRNA screening, molecular and *in silico* analyses with behavioural approaches in a clinically relevant model of metastatic bone-cancer pain in mice, we now show that tumour-induced conditions are associated with a marked dysregulation of 57 miRNAs in sensory neurons corresponding to tumour-affected areas. By establishing protocols for interference with disease-induced miRNA dysregulation in peripheral sensory neurons *in vivo*, we functionally validate six dysregulated miRNAs as significant modulators of tumour-associated hypersensitivity. *In silico* analyses revealed that their predicted targets include key pain-related genes and we identified *Clcn3*, a gene encoding a chloride channel, as a key miRNA target in sensory neurons, which is functionally important in tumour-induced nociceptive hypersensitivity *in vivo*. Our results provide new insights into endogenous gene regulatory mechanisms in cancer pain and open up attractive and viable therapeutic options.

## INTRODUCTION

Various forms of cancer are associated with debilitating pain. Approximately one-third of adults who are actively receiving treatment for cancer and two-thirds of those with advanced malignant disease experience pain. Various types of carcinomas and sarcomas metastasize to skeletal bones and cause spontaneous bone pain, hyperalgesia (exaggerated pain) and allodynia (pain in response to a normally innocuous stimulus), which is accompanied by bone degradation and remodelling of peripheral nerves (Mantyh, 2004; Wacnik et al, 2001). In a large number of clinical cases, cancer-associated pain, particularly the neuropathic component thereof, is resistant to conventional therapeutics or their application is severely limited owing to the widespread side effects (Mantyh, 2006; Portenoy, 1999). In order to develop novel, mechanism-based therapeutic strategies, it is imperative to delineate the cellular and molecular mechanisms underlying cancer-induced pain. Indeed, although tumour-induced pain shares features of inflammatory as well as neuropathic pain, it is clearly distinguished by distinct pathophysiological and mechanistic aspects (Mantyh, 2004; Wacnik et al, 2001).

Non-coding RNAs (ncRNAs), including the more well-studied microRNAs (miRNAs), are emerging as critical modulators of normal cellular functions as well as pathological processes (Huttenhofer & Schattner, 2006; Huttenhofer et al, 2005; Mattick, 2004). Various diseases are associated with unique miRNA expression signatures, which can not only be exploited as diagnostic and prognostic markers, but also reveal deep insights into disease pathology. Moreover, miRNAs can act as ‘master switches’ of the genome to regulate the expression of diverse proteins and orchestrate multiple cellular pathways, thereby harbouring tremendous therapeutic potential.

Recently, neuropathic pain conditions have been suggested to deregulate the expression of miRNAs in pain pathways in profiling studies (Aldrich et al, 2009; Bai et al, 2007; Imai et al, 2011; Kusuda et al, 2011; Poh et al, 2011; von Schack et al, 2011). Moreover, specific miRNAs have been associated with inflammatory pain and the deregulation of ion channel expression in sensory neurons in rodent models of inflammatory and neuropathic pain (Favereaux et al, 2011; Li et al, 2011; Zhao et al, 2010). However, to date, nothing has been reported on the modulation of miRNA expression in conjunction with cancer pain. Moreover, previous studies on miRNA deregulation in pain have mostly remained at the niveau of profiling expression; in contrast, the functional consequences of miRNA deregulation, their downstream targets and the mechanisms by which miRNAs regulate processes modulating nociception have not been resolved.

Both peripheral as well as spinal contributions are of critical importance in understanding cancer pain and developing therapeutic approaches (Gordon-Williams & Dickenson, 2007; Mantyh, 2004). Our key interest is to address plasticity mechanisms at the interface between tumour cells and nociceptive pathway; thereby, mechanisms operational in sensory neurons are of prime interest. This is supported by numerous studies, which have demonstrated changes in the structure as well as the function of sensory neurons in cancer pain states, which are attributed to effects of tumour growth and tumour-associated mediators (Cain et al, 2001; Constantin et al, 2008; Mantyh, 2004).

Starting with a genome-wide screen for miRNAs regulated in sensory neurons of the dorsal root ganglia (DRG) in the state of bone-metastatic pain, we functionally validated a set of prominently regulated miRNAs and report the impact of deregulating their expression in sensory neurons on cancer-associated nociceptive hypersensitivity. Our *in silico* analysis of gene targets of prominently regulated miRNAs not only revealed that several prominent genes encoding known nociceptive mediators, but also uncovered a novel target encoding a chloride channel, which we functionally validated as an important modulator of nociceptive sensitivity. Our results underscore the importance of miRNA regulation in sensory neurons in the context of bone metastatic pain and systematically delineate the potential of ncRNAs as druggable targets for future treatment of cancer-associated pain.

## RESULTS

### Genome-wide identification of miRNAs aberrantly expressed in sensory neurons in the context of bone metastatic pain

Several types of carcinomas and sarcomas metastasize to the bone and bone metastatic pain is the most common form of cancer-related pain (Mantyh, 2006). We therefore used a previously described model of bone metastatic pain based upon unilateral implantation of osteolytic fibrosarcoma cells in the calcaneous bone of paw heel. As we and others have reported previously (Cain et al, 2001; Schweizerhof et al, 2009; Wacnik et al, 2001), tumour growth was associated with the well-described triad of osteolytic tumour spread in the paw tissue, structural changes in sensory nerves, such as hypertrophy and sprouting, and development of intense mechanical hypersensitivity to plantar stimulation of the paw (Schweizerhof et al, 2009). Because tumour cells are known to secrete mediators, which remodel and sensitize sensory neurons of the corresponding DRG mostly L3-L4 in mouse (Rigaud et al, 2008), we addressed how the miRNA repertoire in L3-L4 DRGs changes following peripheral tumour induction.

**Figure 1:**
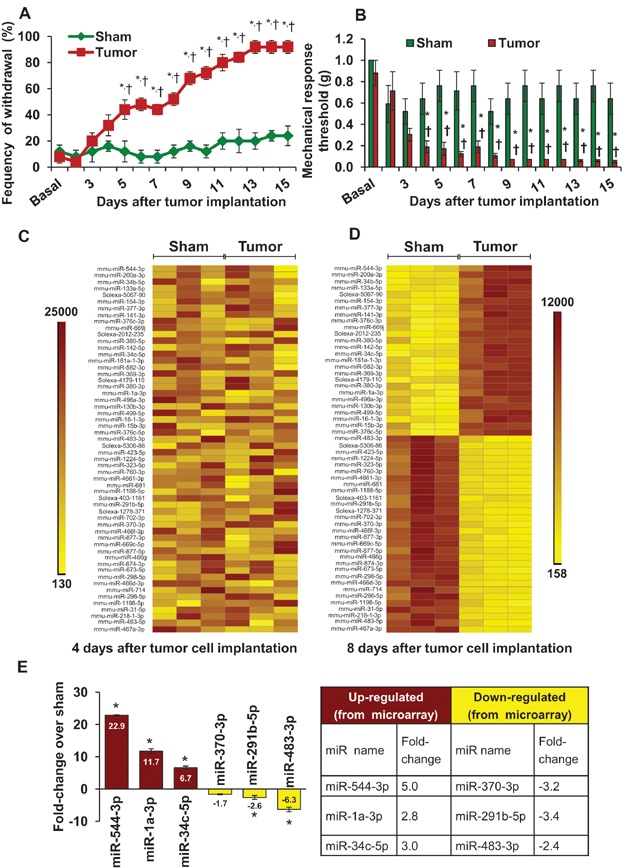
Up- or down-regulation of microRNAs (miRNAs) in sensory neurons of the dorsal root ganglia (DRG) in a model of bone metastases pain **A.** Increase in frequency of paw withdrawal to plantar application of a von Frey filament force of 0.07 g following induction of tumor growth in the calcaneous bone of the heel in mice as compared to sham surgery. * denotes *p* = 0.002 on PID-5, 6, 7 and <0.0001 from PID-8 through 15 as compared to basal and † denotes *p* < 0.001 on PID-5 and <0.0001 from PID-6 through 15 as compared to corresponding data point in the sham group, two-way ANOVA of repeated measures followed by Bonferroni's multiple comparisons *post hoc* test, *n* = at least 6 mice per group.**B.** Mechanical response threshold calculated as von Frey filament strength required to achieve 50% withdrawal frequency. * denotes *p* < 0.001 from PID-4 through 15 as compared to basal and † denotes *p* = 0.004 on PID-5, 6 & 13, 0.006 on PID-7, 9 & 11, 0.005 on PID-8, 0.004 on PID-10, 0.0001 on PID-12 & 14, and 0.003 on PID-15 as compared to corresponding data point in the sham group, two-way ANOVA of repeated measures followed by Bonferroni's multiple comparisons *post hoc* test, *n* = at least 6 mice per group.**C,D.** Heat maps of miRNAs found to be significantly up- or downregulated via microarray analysis in the ipsilateral lumbar DRG of tumor-bearing mice 4 days (C) or 8 days (D) post implantation as compared to sham surgery. Scale indicates expression intensities obtained from the microarray experiment.**E.** Representation of examples of miRNAs showing up- or down-regulation following independent verification with quantitative RT-PCR analyses (left hand panel) and the original data from microarray analysis. *p = 0.001 for miR-544-3p, 0.003 for miR-1a-3p, 0.009 for miR-34c-5p, 0.04 for miR-370-3p, 0.03 for miR-291b-5p and 0.005 for miR-483-3p as compared to sham-treated group, ANOVA followed by *post hoc* Fischer's test, *n* = 3 mice per group.

In contrast to sham-treated mice (saline injection in the calcaneous bone), tumour-bearing mice demonstrated exaggerated sensitivity and aversive withdrawal responses to very low, normally innocuous intensities of mechanical force (*e.g*. 0.07 g). This mechanical hypersensitivity started to become apparent on post-implantation Day 5 (PID-5; *p* = 0.003) and gradually increased over the time course (*p* = 0.011 on PID-6, <0.001 from PID-6 through 15, one-way repeated measures ANOVA followed by Student–Newman–Keuls *post hoc* test). Tumour-induced mechanical hypersensitivity was also apparent upon comparing the 50% response threshold (Fig 1B; *p < 0.01 as compared to corresponding sham control and denotes ^†^*p* < 0.01 as compared to corresponding basal value, two-way ANOVA of repeated measures followed by Bonferroni's multiple comparisons *post hoc* test).

Selecting two different time points after tumour cell implantation, *i.e*. PID-4, when hypersensitivity is just starting to evolve, and PID-8, when significant tumour-mediated hyperalgesia is established, we performed a genome-wide miRNA screen to detect tumour-induced regulation of miRNAs in L3-L4 DRGs. Total RNA was processed and analysed using Illumina miRNA expression arrays pre-spotted with 655 mature miRNAs according to the miRBase version 12.0. Following quantile array normalization to correct for systematic differences between arrays, which do not represent biological variation of interest between experimental groups (Bolstad et al, 2003; Chudin et al, 2006), mean signal intensities for each miRNA were compared across DRGs obtained from tumour-bearing mice (*n* = 3) to the sham group of mice (*n* = 3). Although 86 miRNAs were regulated with 2.0-fold-change, we sought to identify the most prominent changes by focusing on miRNAs which showed at least 2.5-fold-change (up- or downregulation) in expression and with most stringent array and biological replicate standards (see Materials and Methods Section for detailed description) in tumour-bearing mice over sham controls. These were depicted in form of heat plots (Fig 1C and D). Using these criteria, no prominent and consistent changes were observed in miRNA expression between tumour-bearing mice and sham mice on PID-4 (Fig 1C). However, at PID-8, when strong hypersensitivity was established, a subset of 57 miRNAs showed striking changes in expression when compared to sham-treated mice, which were consistent over all three biological replicates (Fig 1D); 26 miRNAs were prominently upregulated and 31 miRNAs were downregulated in tumour-bearing mice as compared to sham-treated mice. Out of these 57 cancer-pain mediated subset of 57 miRNAs, 6 were unannotated and one miRNA namely mmu-miR-197 was withdrawn in subsequent releases of miRBase (Supporting Information Table S1). Microarray profiling data were confirmed with an independent methodology, namely quantitative real-time PCR (qRT-PCR) at PID-8. Results from qRT-PCR analyses largely matched microarray data (examples are shown in Fig 1E), although the extent of regulation tended to be much higher in qRT-PCR analysis than from profiling analyses.

### Manipulating the expression of miRNAs in DRGs *in vivo*

miRNA inhibitors are single stranded oligos having seed sequence complimentarity with targeting miRNA, which inhibits endogenous miRNA function. miRNA mimics on the other hand are synthetic double stranded oligos, which mimics endogenous miRNA expression after entering into the cell. In our hands, cholesterol-conjugated antagomirs were not efficacious in inhibiting expression of miRNAs or their target mRNAs following intrathecal delivery. Therefore, we custom-designed LNA-based miRNA-inhibitors with shortest possible nucleotide lengths without considerably enhancing off-target effects in order to facilitate cellular permeation (Supporting Information Table S2). Mis-match inhibitors were designed as controls in a similar manner by introducing at least four mismatches into respective inhibitor sequence, while making sure not to have any sequence similarity with other mouse miRNA (Supporting Information Table S1). miRNA inhibitors and mis-match inhibitors were delivered intrathecally with an *in vivo* transfection reagent to boost tissue uptake, as described recently (Favereaux et al, 2011). To reverse tumour-mediated down-regulation of candidate miRNAs, we delivered miRNA-mimics, which were custom-designed to carry a 3′-cholesterol conjugation on the passive, passenger strand (to facilitate *in vivo* uptake) and a 3′-FITC conjugation on the guide strand (for visualization).

**Figure 2:**
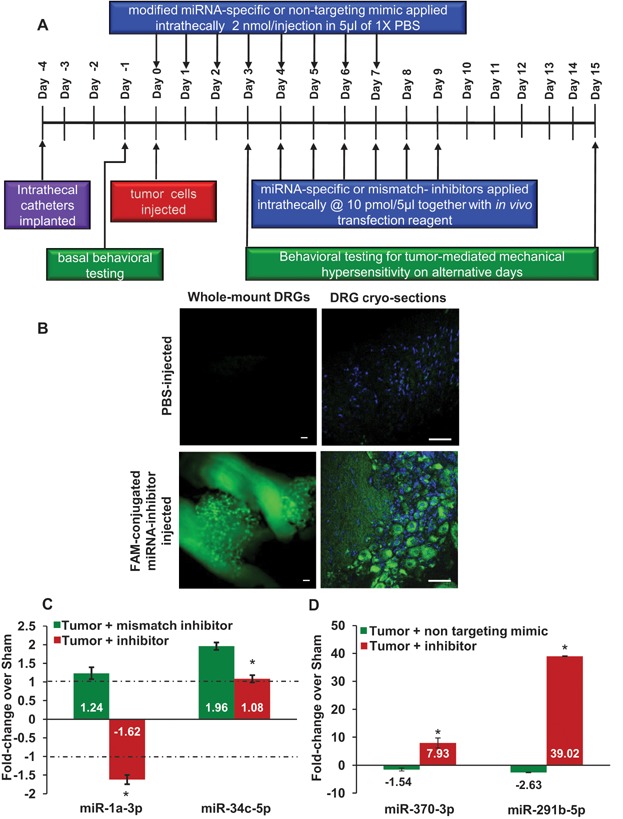
Manipulation of miRNA expression in lumbar DRGs in mice *in vivo* via intrathecal application of miRNA mimics and inhibitors Experimental scheme established in this study which enables effective knockdown/induction of miRNA expression in DRGs *in vivo* and analysis of tumour pain-associated behaviours.Microscopic analyses of whole-mount DRGs or cryosections showing uptake of FAM-conjugated miRNA inhibitors. Scale bar is 50 µm in all panels.Typical examples of qRT-PCR verification of efficacy of miRNA inhibitors in reversing tumour-induced upregulation of miRNAs in ipsilateral DRGs *in vivo*.Typical examples of qRT-PCR verification of efficacy of miRNA mimics in reversing tumour-induced downregulation of miRNAs and inducing overexpression of miRNAs in ipsilateral DRGs *in vivo*. In panel (C), * denotes *p* = 0.02 for miR-1a-3p, 0.04 for miR-34c-3p as compared to corresponding mismatch inhibitor and in panel (D), * denotes *p* = 0.001 for miR-370-3p and <0.0001 for miR-291b-5p as compared to non-targeting mimic, ANOVA followed by *post hoc* Fischer's test, *n* = 3 per group.

We implanted chronic intrathecal catheters, which permitted delivery of multiple doses of miRNA inhibitors or mimics, adapting regimens previously described for delivering small interfering RNAs to DRGs in rats *in vivo* (Whitehead et al, 2009; Wu et al, 2010). Based on previous observations that intrathecally delivered fluorescence probe-tagged short-oligonucleotides can be detected in the DRGs only up to 24 h following injection (Layzer et al, 2004; Mook et al, 2010), we performed multiple administrations of either inhibitor or mimic intrathecally at the lumbar L3-L4 DRG level *in vivo* at 24 h intervals. As shown in the Fig 2, panel B, intrathecally delivered FAM-tagged miRNA-inhibitors (2 nmol/injection in 5 µl volume followed by 5 µl saline) could be visualized in whole mount DRGs at 24 h after a single bolus application; in images at higher magnification, fluorescence was detected in the cytoplasm of the labelled cells throughout the DRG (Fig 2, panel B). Quantitative analyses are difficult owing to the low level of fluorescence signals detected from the single FAM molecule tagged per inhibitor molecule; however, upon confocal analysis of sections derived from DRGs, 73% of DRG neurons showed an uptake of FAM-conjugated inhibitors. In contrast, confocal analysis on spinal L3/L4 segment sections did not reveal uptake in the spinal dorsal horn of the same animals (Supporting Information Fig 1).

Moreover, functionality of intrathecally delivered inhibitors or mimics was validated via qRT-PCR-based expression analysis on lumbar L3, L4 and L5 DRGs 24 h after injection (examples with knockdown of miR-1a-3p and miR-34c-3p with their respective inhibitors in comparison with respective mismatch inhibitors is shown in Fig 2, panel C; *p* < 0.05 two tailed *t*-test). Along the same lines, DRGs from mice injected intrathecally with miRNA mimics showed a strong expression of targeted miRNAs in comparison to corresponding non-targeting mimics (examples with overexpression of miR-370-3p and miRNA-291b-5p with their respective mimics in comparison with non-targeting-controls is shown in Fig 2, panel D; *p* < 0.05 two tailed *t*-test).

These approaches for reciprocal modulation of the expression of deregulated candidate miRNAs in DRG *in vivo* thus paved the way for functional experiments in the context of tumour-pain model.

### Elucidating the functional significance of tumour-associated upregulation of miRNAs in bone metastatic pain

We then chose a set of six candidate miRNAs for *in vivo* functional analysis, which had shown interesting patterns of expression changes in tumour-bearing mice. In all of the behavioural analyses described below, * denotes *p* ≤ 0.05 as compared to basal values, † denotes *p* ≤ 0.05 relative to corresponding mismatch inhibitor or mimic for each particular day of analysis and ‡ denotes *p* ≤ 0.05 relative to vehicle treated group for particular day of analysis. The statistical analyses were performed by employing two-way ANOVA of repeated measures followed by Bonferroni's multiple comparisons *post hoc* test.

**Figure 3:**
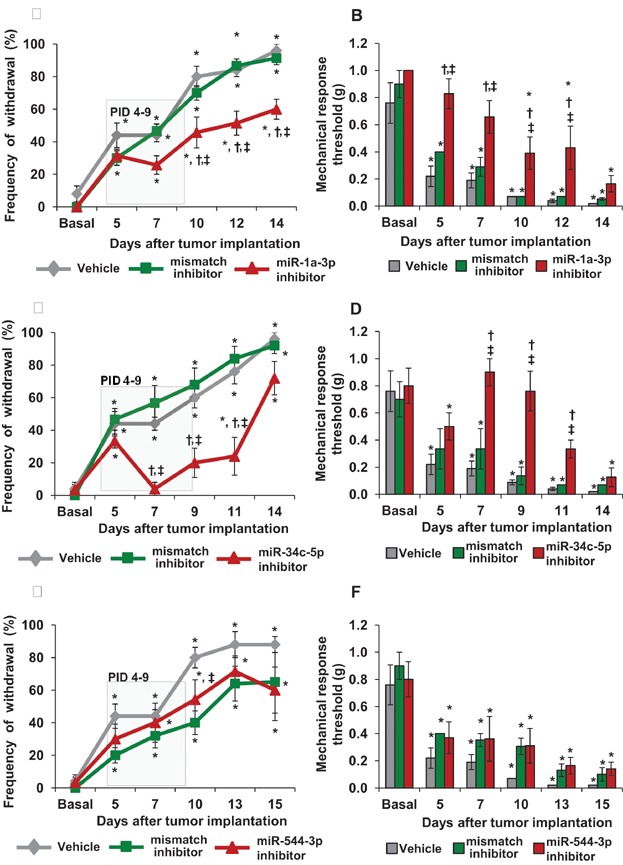
Functional validation of miRNAs upregulated in DRGs in tumor bearing mice with respect to tumor-induced mechanical hypersensitivity Change in frequency of paw withdrawal to plantar application of a von Frey filament force of 0.07 g following induction of tumor growth in the calcaneous bone of the heel in mice receiving intrathecally delivered miR-1a-3p inhibitor (red symbols) or the corresponding mismatch inhibitors (green symbols) or vehicle (grey symbols). * denotes *p* < 0.0001 on PID-5 through 15 in the vehicle, mismatch inhibitor and miR-1a-3p inhibitor groups as compared to basal; † denotes *p* = 0.007 on PID-10 and <0.0001 on PID-12 & 14 as compared to corresponding data point in the mismatch inhibitor group; ‡ denotes *p* < 0.0001 on PID-10, 12 & 14 as compared to corresponding data point in the vehicle group.Mechanical response thresholds calculated as von Frey filament strength required to achieve 50% withdrawal frequency following induction of tumor growth in the calcaneous bone of the heel in mice receiving intrathecally delivered miR-1a-3p inhibitor (red bars) or the corresponding mismatch inhibitors (green bars) or vehicle (grey bars). * denotes *p* < 0.0001 from PID-5 through 14 in the vehicle and mismatch inhibitor groups and on PID-10, 12, 14 in the miR-1a-3p-inhibitor groupas compared to basal; † denotes *p* = 0.004 on PID-5, 0.003 on PID-7, 0.05 on PID-10 and 0.01 on PID-12 as compared to corresponding data point in the mismatch inhibitor group; ‡ denotes *p* < 0.0001 on PID-5, 0.0002 on PID-7, 0.05 on PID-10, and 0.004 on PID-12 as compared to corresponding data point in the vehicle group.Change in frequency of paw withdrawal to plantar application of a von Frey filament force of 0.07 g following induction of tumor growth in the calcaneous bone of the heel in mice receiving intrathecally delivered miR-34c-5p inhibitor (red symbols) or the corresponding mismatch inhibitor (green symbols) or vehicle (grey symbols). * denotes *p* < 0.0001 on PID-5 through 14 in the vehicle and mismatch inhibitor groups, 0.006 on PID-5 and < 0.0001 on PID-14 for miR-34c-5p- inhibitor group as compared to basal; † denotes *p* < 0.0001 on PID-7, 9 & 11 as compared to corresponding data point in the mismatch inhibitor group; ‡ denotes *p* < 0.0001 on PID-7, 9 & 11 as compared to corresponding data point in the vehicle group.Mechanical response thresholds calculated as von Frey filament strength required to achieve 50% withdrawal frequency following induction of tumor growth in the calcaneous bone of the heel in mice receiving intrathecally delivered miR-34c-5p inhibitor (red bars) or the corresponding mismatch inhibitors (green bars) or vehicle (grey bars). * denotes *p* = 0.0003 on PID-7 & 9, < 0.0001 on PID-11 & 14 in the mismatch-inhibitor group; 0.0018 on PID-5 and < 0.0001 on PID-14 in the miR-34c-5p-inhibitor group and 0.0001 on PID-5 through 15 in the vehicle group; † denotes *p* < 0.0001 on PID-7, 0.0009 on PID-9, 0.05 on PID-11 as compared to corresponding data point in the mismatch inhibitor group; and ‡ denotes *p* < 0.0001 on PID-7 & 9, 0.0518 on PID-11 as compared to corresponding data point in the vehicle group.Change in frequency of paw withdrawal to plantar application of a von Frey filament force of 0.07 g following induction of tumor growth in the calcaneous bone of the heel in mice receiving intrathecally delivered miR-544-3p inhibitor (red symbols) or the corresponding mismatch inhibitors (green symbols) or vehicle (grey symbols). * denotes *p* < 0.0001 from PID-3 through PID-14 in vehicle, mismatch-inhibitor and miR-544-3p inhibitor groups.Mechanical response thresholds calculated as von Frey filament strength required to achieve 50% withdrawal frequency following induction of tumor growth in the calcaneous bone of the heel in mice receiving intrathecally delivered miR-544-3p inhibitor (red bars) or the corresponding mismatch inhibitors (green bars) or vehicle (grey bars). * denotes *p* < 0.0001 from PID-3 through PID-14 in mismatch-inhibitor and miR-544-3p inhibitor groups. In all panels statistical significance was tested by two-way ANOVA of repeated measures followed by Bonferroni's multiple comparisons *post hoc* test, n = at least 6 mice per group. The experimental scheme employed is the same as described in Fig. 2A. The square box represents the time-course of miRNA-inhibitor or mismatch-inhibitor or vehicle application.

Because we observed that miR-1a-3p is upregulated by >10-fold in the DRG at PID-8 following tumour induction in the calcaneous bone, we asked whether reversing this pathophysiological induction influences tumour-associated hyperalgesia. Mice were intrathecally injected with either miR-1a-3p-inhibitor or a mismatch inhibitor (as described above) at a concentration 10 pmol in a total of 10 µl volume per injection every 24 h starting on PID-4 until PID-9 (see injection scheme in Fig 2, panel A). Although both cohorts of mice developed significant mechanical hypersensitivity following tumour induction as compared to basal behaviour, the magnitude of mechanical hypersensitivity was significantly lesser in mice treated with miR-1a-3p-inhibitor as compared to mice treated with the corresponding mismatch inhibitor (Fig 3, panel A). This was also evident by analysing the 50% response threshold (Fig 3, panel B) or the area under the curve (AUC) of stimulus–response frequency curves for von Frey forces ranging from 0.02 to 1.0 g (Supporting Information Fig 2, panel A).

To study miRNA-34c-5p, which we found to be upregulated by sevenfold in the DRGs at PID-8 following tumour induction, we designed an inhibitor of 14 nucleotides long to target miR-34c-5p specifically, *i.e*. without affecting other miR-34c family members, and a corresponding mismatch control inhibitor (Supporting Information Table S1). Mice treated with the mismatch inhibitor developed significant plantar mechanical hypersensitivity to von Frey force of 0.07 g (Fig 3, panel C), with the same time course and amplitude as untreated mice (Fig 1A). In contrast, mice, which received miR-34c-5p inhibitor from PID-4 to PID-9 did not show significant tumour-associated mechanical hypersensitivity to 0.07 g force on days PID-7, PID-9 and PID-11 (Fig 3, panel C). When the data were analysed as response thresholds (Fig 3, panel D) or AUC (Supporting Information Fig 2, panel B) of stimulus–response frequency curves for von Frey strength ranging from 0.02 to 1.0 g, treatment with the miR-34c-5p inhibitor was found to impart a significant level of protection against tumour-associated mechanical hypersensitivity.

However, not all of the miRNAs which were observed to be upregulated in DRGs following tumour induction emerged as functionally relevant in the context of tumour-associated mechanical hypersensitivity. For example, inhibiting tumour-associated upregulation of miR-544-3p, which was a highly regulated candidate miRNA from the screen, resulted in no significant change in the magnitude of tumour-associated mechanical hyperalgesia when compared to corresponding controls (Fig 3, panels D, E and Supporting Information Fig 2, panel C; *p* > 0.05 at all time points tested).

### Elucidating the functional significance of tumour-associated downregulation of miRNAs in bone metastatic pain

**Figure 4:**
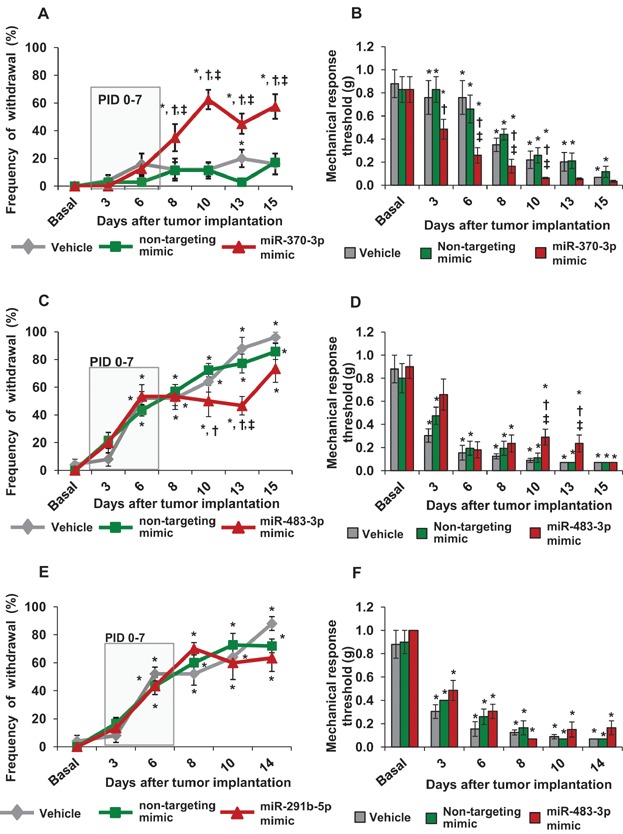
Functional validation of miRNAs downregulated in DRGs in tumor bearing mice with respect to tumor-induced mechanical hypersensitivity Change in frequency of paw withdrawal to plantar application of a von Frey filament force of 0.02 g following induction of tumor growth in the calcaneous bone of the heel in mice receiving intrathecally delivered miR-370-3p mimic (red symbols) or non-targeting mimic (green) or vehicle (grey symbols). * denotes *p* = 0.0192 on PID-13 in the vehicle group and <0.0001 on PID-8 through 15 in the miR-370-3p-mimic group as compared to basal; † denotes *p* = 0.0013 on PID-8 and <0.001 on PID-10, 13 & 15 as compared to corresponding data point in the non-targeting mimic group; ‡ denotes *p* = 0.0003 on PID-8, <0.0001 on PID-10 & 15 and 0.0044 on PID-13 as compared to corresponding data point in the vehicle group.Mechanical response thresholds calculated as von Frey filament strength required to achieve 80% withdrawal frequency following induction of tumor growth in the calcaneous bone of the heel in mice receiving intrathecally deliveredmiR-370-3p mimic (red bars) or non-targeting mimic (green bars) or vehicle (grey bars). * denotes *p* = 0.0291 on PID-8, 0.05 on PID-10, 0.0192 on PID-13 and 0.05 on PID-15 in the vehicle group, 0.04 on PID-8, 0.002 on PID-10, 0.05 on PID-13 & 15 in the non-targeting mimic group; † denotes *p* = 0.0013 on PID-3, <0.0001 on PID-6, 8 & 10 as compared to corresponding data point in the non-targeting mimic group; ‡ denotes *p* = 0.0013 on PID-6 and <0.0001 on PID-8, 10 & 13 as compared to corresponding data point in the vehicle group.Change in frequency of paw withdrawal to plantar application of a von Frey filament force of 0.07 g following induction of tumor growth in the calcaneous bone of the heel in mice receiving intrathecally delivered miR-483-3p mimic (red symbols) or non-targeting mimic (green) or vehicle (grey symbols). * denotes *p* = 0.0008 on PID-6, 0.0002 on PID-8 & <0.0001 on PID-10, 13, 15 in vehicle, non-targeting mimic and miR-483-3p mimic groups as compared to basal; † denotes *p* = 0.0206 on PID-10 & 0.0074 on PID-13 as compared to corresponding data point in the non-targeting mimic group; and ‡ denotes *p* = 0.0074 on PID-13 as compared to corresponding data point in the vehicle group.Mechanical response thresholds calculated as von Frey filament strength required to achieve 50% withdrawal frequency following induction of tumor growth in the calcaneous bone of the heel in mice receiving intrathecally deliveredmiR-483-3p mimic (red bars) or non-targeting mimic (green bars) or vehicle (grey bars). * denotes *p* < 0.0001 from PID-3 through 15 in vehicle, non-targeting-mimic groups and miR-483-3p-mimic groups as compared to basal; † denotes *p* = 0.005 on PID-10 & 0.05 on PID-13 as compared to corresponding data point in the non-targeting mimic group; and ‡ denotes *p* = 0.031 on PID-10, 0.022 on PID-13 as compared to corresponding data point in the vehicle group.Change in frequency of paw withdrawal to plantar application of a von Frey filament force of 0.07 g following induction of tumor growth in the calcaneous bone of the heel in mice receiving intrathecally delivered miR-291b-5p mimic (red symbols) or non-targeting mimic (green) or vehicle (grey symbols). * denotes *p* < 0.0001 from PID-3 through PID-14 in vehicle, non-targeting-mimic and miR-291b-5p-mimic groups.Mechanical response thresholds calculated as von Frey filament strength required to achieve 50% withdrawal frequency following induction of tumor growth in the calcaneous bone of the heel in mice receiving intrathecally deliveredmiR-291b-5p mimic (red bars) or non-targeting mimic (green bars) or vehicle (grey bars). * denotes *p* < 0.0001 from PID-3 through PID-14 in vehicle, non-targeting-mimic and miR-291b-5p-mimic groups. In all panels, statistical significance was tested by two-way ANOVA of repeated measures followed by Bonferroni's multiple comparisons *post hoc* test, n = at least 6 mice per group. The experimental scheme employed is the same as described in Fig. 2A. The square box represents the time-course of miRNA-mimic or non-targeting-mimic or vehicle application.

We then proceeded to investigate the functional significance of candidate miRNAs that were downregulated in tumour conditions by using mimics to counteract tumour-induced miRNA downregulation in DRGs. Either a miRNA-specific or a non-targeting mimic was applied starting the Day 0 of tumour induction intrathecally until PID-8 at 2 nmol/injection in 5 µl PBS followed by 5 µl saline flush (see injection scheme in Fig 2, panel A). Interestingly, mice treated with a miR-370-3p-mimic developed significantly more tumour-induced mechanical hypersensitivity to a von Frey force of 0.02 g than mice treated with a non-targeting control mimic (Fig 4, panel A; *p* < 0.05). This was also evident upon comparing response thresholds (Fig 4, panel B) or AUC (Supporting Information Fig 3, panel A) of stimulus–response frequency curves over von Frey forces 0.02–1 g. Thus, counteracting tumour-induced downregulation of miR-370-3p and overexpressing it in DRGs led to exaggerated tumour-associated pain, indicating that downregulation of miR-370-3p constitutes an endogenous defense mechanism against tumour-pain.

In contrast, a different functional role was observed for miR-483-3p in this model of tumour-pain. Although mice treated with a miR-483-3p-mimic developed significant mechanical hypersensitivity to 0.07 g of mechanical force following tumour induction as compared to basal behaviour, the magnitude of mechanical hypersensitivity observed was significantly lesser as compared to that seen in mice treated with a non-targeting control mimic (Fig 4, panel C). Analysis of response thresholds (Fig 4, panel D) or AUC (Supporting Information Fig 3, panel B) of stimulus–response frequency curves for von Frey forces ranging from 0.02–1.0 g revealed similar results. Thus, tumour-induced downregulation of miRNA-483-3p contributes to nociceptive hypersensitivity.

Finally, we observed that reversing the down-regulation of miRNA-291b-5p, another candidate deregulated miRNA from profiling analyses, resulted in no significant change in the magnitude of tumour-associated mechanical hyperalgesia when compared to corresponding controls (Fig 4, panels E, F and Supporting Information Fig 3, panel C, *p* > 0.05).

### Addressing mechanisms underlying miRNA-mediated modulation of tumour-associated hypersensitivity via analysis of mRNA targets

Having functionally validated several candidate miRNAs, which emerged from our genome-wide screen, we then set out to investigate mechanisms underlying their role in tumour-associated mechanical hypersensitivity. Using *in silico* algorithms for prediction of mRNA targets of miRNAs, we noted that several important modulators of neuronal excitability and nociceptive sensitivity are putatively modulated by miRNAs, which emerged from our screen. Due to very large volume of these *in silico* data sets, we focus here on one particular deregulated miRNA to present a comprehensive overview in the context of tumour pain, starting from *in silico* prediction of mRNA targets, qRT-PCR validation of predictions, promoter analysis of one specific key mRNA to verify mRNA–miRNA pairing, analysis of it's modulation *in vivo* and finally, *in vivo* functional analysis of the mRNA target in tumour-associated pain. For this purpose, we chose miRNA-1a-3p as our prototypic miRNA for detailed mechanistic analysis for various reasons (see Discussion Section). As the first step, we followed an unbiased and comprehensive approach to predict the mRNAs targets for miRNA-1a-3p by adapting 14 different state-of-art algorithms (Supporting Information Table S3), which utilize different parameters, such as seed region complementarily and thermodynamic stability to predict the targets of a given miRNA. Reasoning that a transcript predicted as a target by several algorithms is likely to be a bona-fide, biologically significant target in terms of miRNA–mRNA pairing, we derived a condensed list of 53 candidate mRNA targets for the miRNA-1a-3p.

**Figure 5:**
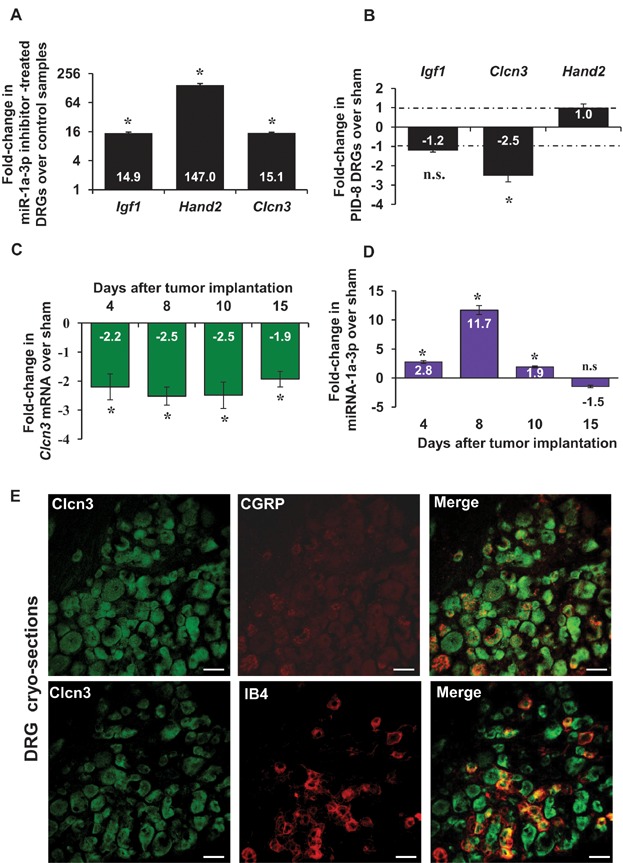
Analysis of miR-1a-3p target genes in the context of tumor-induced hypersensitivity in the bone metastatic pain model QRT-PCR analysis demonstrating induction of predicted miR-1a-3p target genes in lumbar DRGs *in vivo* following suppression of miR-1a-3p expression via inhibitor delivery intrathecally *in vivo*. * denotes *p* <:0.0001 as compared to mismatch-inhibitor group.QRT-PCR analysis representing changes in expression of miR-1a-3p target genes in ipsilateral lumbar DRGs *in vivo* at day 8 in the bone metastases model. * denotes *p* = 0.041 as compared to sham group.Time course of tumor-induced downregulation of *Clcn3* expression in ipsilateral lumbar DRGs via QRT-PCR analysis. * denotes *p* = 0.002 on PID-4, 0.003 on PID-8, 0.05 on PID-10 and 0.04 on PID-15 as compared to sham group.Time course of tumor-induced changes in miR-1a-3p expression in ipsilateral lumbar DRGs via QRT-PCR analysis. * denotes *p* = 0.04 on PID-4, 0.002 on PID-8, and 0.04 on PID-10 as compared to sham group.In panels A, B, C &D statistical P value was calculated by ANOVA followed by *post hoc* Fischer's test, *n* = 3 mice per group.Immunohistochemical analysis of expression of Clcn3 protein in mouse DRG and colabeling with isolectin-B4-binding (IB4) non-peptidergic nociceptors and peptidergic (CGRP-positive) nociceptors. Scale bars represent 50 µm.

To comprehensively exclude false positives or targets that may be regulated only modestly, we directly analysed the impact of miRNA-1a-3p inhibition on the expression of all 53 target mRNAs in the DRG *in vivo*. Using a nanostring device, which digitally quantifies a wide set of mRNA transcripts starting from a very low quantity of tissue, we analysed RNA samples obtained from DRGs of mice which received intrathecal injections of 10 pmol of miRNA-1a-3p inhibitor or mismatch inhibitor *in vivo* every 24 h with *in vivo* transfection reagent from PID-4 to PID-9. Following qRT-PCR-based confirmation of the inhibition of miRNA-1a-3p by it's specific inhibitor, we then analysed the expression of 53 predicted targets in relation to that of five housekeeping genes, namely clathrin, heavy polypeptide (*Cltc*), glyceraldehyde-3-phosphate dehydrogenase (*Gapdh*), glucuronidase beta (*Gusb*), hypoxanthine guanine phosphoribosyl transferase (*Hprt*) and tubulin, beta 5 class I (*Tubb5*). Amongst these, the expression of 3 targets, namely insulin-like growth factor 1 (*Igf1*), heart and neural crest derivatives expressed transcript 2 (*Hand2*) and Chloride channel 3 (*Clcn3*) (Fig 5, panel A), was strongly enhanced upon suppression of miRNA-1a-3p expression, suggesting miRNA–mRNA pairing.

### Dysregulation of *Clcn3* expression in the DRG in bone metastatic pain and it's relation to miR-1a-3p

Owing to the potential impact of chloride channels on neuronal excitability, we were particularly interested in characterizing *Clcn3* as a new target of miR-1a-3p. Based upon the above results, we were intrigued by the question whether modulation of *Clcn3* expression directly contributes to the functional role, which we observed for miRNA-1a-3p in the bone metastatic model (above). Interestingly, qRT-PCR analysis on DRGs from tumour-bearing mice revealed that at PID-8, a time point at which we had initially observed that miRNA-1a-3p is upregulated (Fig 1, panel E), the expression levels of *Clcn3* mRNA are significantly lower than levels in basal state or sham controls (Fig 5, panels B and C; two-tailed *t*-test assuming equal variances). In contrast, expression of *Igf1* and *Hand2* was not significantly altered at PID-8 (Fig 5, panel B). To confirm the change in *Clcn3* mRNA expression in the DRGs of tumour-bearing mice, we performed a qRT-PCR-based analysis of the time-course of regulation at different time points after tumour cell implantation as compared with sham controls at each time point. We observed that *Clcn3* is significantly and consistently downregulated starting from PID-4 until PID-15 (Fig 5, panel C), which is in complete agreement with the time-course of development of tumour-induced mechanical hyperalgesia (Fig 1A). Moreover, this fits to the time-course of miR-1a-3p upregulation in ipsilateral DRGs following tumour induction (Fig 5, panel D, two-tailed *t*-test assuming equal variances). Taken together with the *ex vivo* analyses, these experiments underscore the tight reciprocal association between the expression of miRNA-1a-3p and *Clcn3* and established their *in vivo* validity as a miRNA–mRNA modulatory pair in DRGs of tumour-bearing mice.

Using a previously characterized antibody directed against mouse Clcn3 protein (Matsuda et al, 2008), we detected Clcn3 immunoreactivity in a majority of DRG neurons (Fig 5, panel E). Control sections stained with secondary antibodies alone did not reveal auto fluorescence (Supporting Information Fig 4). Co-labelling experiments indicated anti-Clcn3 immunoreactivity overlapped with CGRP expression and isolectin-B4-binding, which constitute markers of peptidergic and non-peptidergic nociceptive neurons, respectively (Fig 5, panel E).

### Validation of *Clcn3* as a target for miR-1a-3p

**Figure 6:**
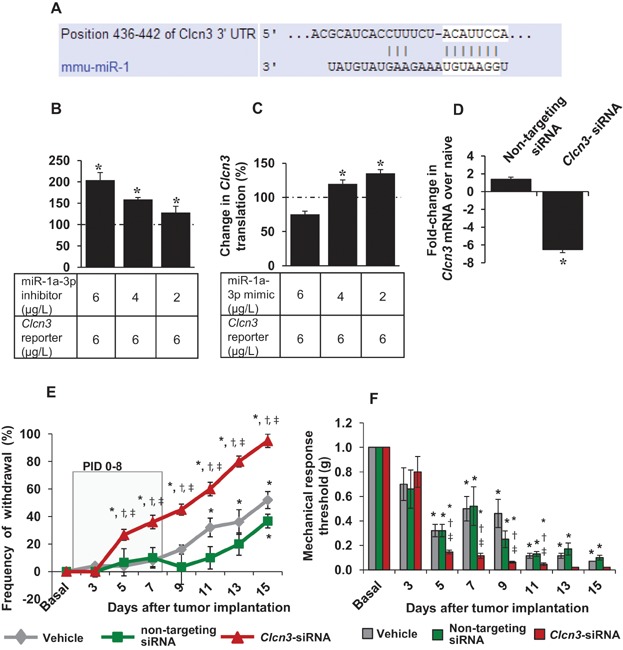
*In vitro* and *in vivo* validation of *Clcn3* as a miR-1a-3p target gene and its functional contribution to tumor-induced mechanical hypersensitivity in the bone metastatic pain model employed Binding sites for miR-1a-3p (mmu-miR-1) on the 3' untranslated region (UTR) of the mouse *Clcn3* gene.Luciferase-reporter based assay in HEK293 cells demonstrating changes in translation of the *Clcn3* gene following suppression of miR-1a-3p expression via graded delivery of the specific inhibitor. *p = 0.04, 0.02 and 0.05 for 6, 4 & 2 µg/L miR-1a-3p inhibitor groups respectively as compared to control, Student's *t*-test, *n* = 3 independent experiments.Luciferase-reporter based assay in HEK293 cells demonstrating changes in translation of the *Clcn3* gene following suppression or induction of miR-1a-3p expression via graded delivery of the specific mimic. *p = 0.03, & 0.02 for 4 & 2 µg/L miR-1a-3p inhibitor groups respectively as compared to control, Student's *t*-test, *n* = 3 independent experiments.QRT-PCR analysis demonstrating significant knockdown of *Clcn3* expression in lumbar DRG following intrathecal delivery of a siRNA directed against *Clcn3* or the corresponding control non-targeting siRNA, *p = 0.001 as compared to naive group, ANOVA followed by *post hoc* Fischer's test, *n* = 3 mice per group.Change in frequency of paw withdrawal to plantar application of von Frey filament forces of 0.02 g following induction of tumor growth in the calcaneous bone of the heel in mice receiving intrathecally delivered siRNA directed against *Clcn3* (red symbols) or the corresponding non-targeting siRNA (green symbols) or vehicle (grey symbols). * denotes *p* = 0.0006 on PID-11, and <0.0001 on PID-13 & 15 in the vehicle group, <0.0001 on PID-15 in the non-targeting siRNA group, 0.0037 on PID-5, and <0.0001 on PID-7 through 15 for the *Clcn3*-siRNA group as compared to basal, † and ‡ denotes *p* = 0.0202 on PID-5, 0.0011 on PID-7 and <0.0001 on PID-9, 11, 13 & 15 as compared to corresponding data point in the non-targeting siRNA or vehicle groups respectively. The square box represents the time-course of *Clcn3*-siRNA or non-targeting-siRNA or vehicle application.Mechanical response thresholds calculated as von Frey filament strength required to achieve 80% withdrawal frequency following induction of tumor growth in the calcaneous bone of the heel in mice receiving intrathecally deliveredsiRNA directed against *Clcn3* (red bars) or the corresponding non-targeting siRNA (green bars) or vehicle (grey bars). * denotes *p* = 0.0003 on PID-5 & 9, 0.0233 on PID-7, and <0.0001 on PID-11, 13 & 15 in the vehicle group, 0.0003 on PID-5, 0.002 on PID-7 and <0.0001 on PID-9, 11, 13 & 15 for the non-targeting-siRNA group and <0.0001 from PID-5 through PID-15 for the Clcn3-siRNA group as compared to basal; † denotes *p* = 0.0122 on PID-5, 0.0024 on PID-7, 0.0127 on PID-9, 0.05 on PID-11 as compared to corresponding data point in the non-targeting-siRNA group and ‡ denotes *p* = 0.0122 on PID-5, 0.0002 on PID-7, 0.0029 on PID-9, and 0.0401 on PID-11 as compared to corresponding data point in the vehicle group.

*In silico* analysis indicated that miR-1a-3p can bind to the 3 prime untranslated region (3′UTR) of the mouse *Clcn3* gene (Fig 6, panel A). To confirm that miRNA-1 is able to functionally bind to 3′ UTR of *Clcn3* and modulate it's expression, we constructed a luciferase reporter vector in which the 3′UTR of *Clcn3* mRNA was fused to the luciferase coding sequence under pGK promoter in dual luciferase vector (pmiRGLO vector). In a HEK293 cell-based heterologous expression system, either miR-1a-3p or mismatch inhibitors were co-transfected with the *Clcn3* reporter vector. In a parallel experiment, either miR-1a-3p mimic or non-targeting mimic were co-transfected with the *Clcn3* reporter vector and luciferase expression was measured 48 h thereafter. Expression of miR-1a-3p inhibitor dose-dependently increased the translation of luciferase protein from the *Clcn3* reporter construct, whereas expression of the miR-1a-3p mimic exerted the opposite effect (Fig 6, panels B and C, *p* < 0.05, as compared to control samples, two tailed *t*-test). These results demonstrate that miR-1a-3p directly targets the *Clcn3* 3′UTR region to modulate its expression.

### Knock-down of *Clcn3* leads to enhanced tumour-induced mechanical hypersensitivity

Finally, we tested the functional significance of knocking down *Clcn3* expression in DRG in the context of tumour-induced hypersensitivity. A pool of siRNAs designed against *Clcn3* mRNA (*Clcn3*-siRNA) or non-targeting siRNA (control-siRNA) were applied intrathecally (5 µl at 1.2 µg/µl siRNA with *in vivo* transfection reagent), which led to a significant knock-down of *Clcn3* expression within 48 h of a single dose of *Clcn3*-siRNA as compared to control-siRNA (Fig 6, panel D, *p* < 0.05, as compared to control samples, two tailed *t*-test). For behavioural analysis in the tumour pain model, siRNAs were injected from PID-0 until PID-8 every 24 h and the development of tumour-mediated mechanical hypersensitivity was monitored using calibrated von Frey filaments from PID-3 until PID-15 on every alternate day. Interestingly, a knockdown of *Clcn3* expression in DRG was associated with exaggerated mechanical hypersensitivity to plantar application of von Frey stimuli in tumour-bearing mice. Whereas control siRNA-treated mice did not develop any hypersensitivity to extremely low magnitude of mechanical force, such as in response to the von Frey filament exerting 0.02 g force until 11 days after tumour-induction, *Clcn3*-siRNA-treated mice showed significant hypersensitivity to 0.02 g from PID-5 onwards (Fig 6, panel E). Moreover, starting from PID-5, mice treated with *Clcn3*-siRNA demonstrated significantly stronger decrease in the mechanical threshold than control-siRNA-treated mice (Fig 6, panel F). Thus downregulating expression of *Clcn3* in the DRG exerted the same functional effect on tumour-associated mechanical hypersensitivity as inhibition of miR-1a-3p, again emphasizing the *in vivo* significance of miR-1a-3p-*Clnc3* as a miRNA–mRNA regulatory pair.

To investigate whether *Clcn3* is also involved in the modulation of basal mechanical sensitivity, we performed *Clcn3* knockdown in L3-L4 DRGs of wild-type mice in the absence of tumour induction. While mice receiving control-siRNA showed no significant alterations in responses to von Frey stimuli, mice treated with the *Clcn3*-siRNA developed significant mechanical hypersensitivity (responses to 0.02 g stimulation are shown in Supporting Information Fig 5, panel A). Analysis of mechanical response thresholds revealed similar results (Supporting Information Fig 5 panel B). However, the magnitude of increase in the mechanical sensitivity upon *Clcn3* knock-down in naïve mice was lesser in magnitude than when compared to magnitude of the effect of *Clcn3*-siRNA on tumour-mediated hyperalgesia, consistent with a regulation of *Clcn3* expression in tumour states.

## DISCUSSION

Despite recent advances, cancer pain remains a major challenge for clinicians and basic scientists and there is an urgent demand for the development of specific mechanism-based therapies. Using a comprehensive approach combining genome-wide miRNA screening, molecular and *in silico* analyses with behavioural approaches in a clinically relevant model of metastatic bone-cancer pain, we now show that tumour-induced hypersensitivity is associated with a dysregulation of miRNA expression in sensory neurons corresponding to tumour-affected areas. This is the first study profiling genome-wide miRNA expression and their functional importance in the development and maintenance of tumour-mediated chronic pain. Furthermore, whereas previous studies on the involvement of miRNAs in other forms of pain disorders, *e.g*. neuropathic pain, have either addressed miRNA profiling without functional analysis or have focussed on a single target gene modulated by a miRNA (Aldrich et al, 2009; Bai et al, 2007; Favereaux et al, 2011), the present study spans multiple levels of analysis starting with a genome-wide identification of the miRNA regulatory network of cancer pain-associated miRNAs in sensory neurons, their functional validation *in vivo* all the way to the identification, molecular characterization and functional validation of a novel target gene.

Our *in vivo* analyses revealed that the expression of 57 miRNAs amongst the 615 tested mouse miRNAs is dysregulated in sensory neurons of the DRG directly under the influence of tumour growth in their innervation territory, representing a novel and intriguing aspect of tumour–nerve interactions. Interestingly, miRNA dysregulation only became evident when pronounced tumour-induced hypersensitivity was established, but did not manifest at the beginning stages (*e.g*. 4 days post-implantation). This suggests that early periods of tumour pain likely result from local interactions between tumour cells and nerves, which lead to sensitization of nerve afferents, and that miRNA dysregulation and the resulting changes in the expression of a multitude of genes in sensory neurons rather contribute to the maintenance and long-term nature of tumour pain. This study was based upon a model involving tumour-induced remodelling of bone and ensuing mechanical hypersensitivity in the adjacent skin. It will be interesting in future studies to compare miRNA expression profiles with models based upon direct injection of tumour cells in the skin to work out the component of bone pain.

In contrast to tissues such as liver, heart, blood vessel and the lymphatic system, amongst others, which are amenable to modulation via exogenously delivered miRNA modulators (Bernardo et al, 2012; Krutzfeldt et al, 2005, 2007; Muramatsu et al, 2013; Pullamsetti et al, 2012; Yigit et al, 2012), the nervous system is notoriously difficult to target (Long & Lahiri, 2012). To disrupt the pathophysiological induction or downregulation of miRNA in DRGs *in vivo*, we established effective protocols for intrathecal delivery of miRNA inhibitors or mimics and demonstrated the efficacy of selective manipulations in miRNA expression *in vivo*. Our behavioural analysis in the bone-metastases model indicated that inhibiting the tumour-induced upregulation of miR-1a-3p or miR-34c-5p, but not of miRNA-544-3p, in sensory neurons markedly attenuated tumour-mediated hyperalgesia. Furthermore, reversing pathophysiological decrease of miR-483-3p, but not of miR-291b-5p, attenuated tumour-mediated hyperalgesia. In contrast, augmenting the expression of miR-370-3p in DRGs led to exaggerated tumour-mediated hyperalgesia. Thus, the approach established and described in this study provides proof-of-principal that it is effective and valuable in elucidating the functional contributions of miRNAs. An intriguing observation is that even though injection of miRNA modulators was stopped at PID-9, the effect persisted until PID-15. As it has been shown that short-oligonucleotides can be detected in the DRGs up to 24 h following injection (Layzer et al, 2004; Mook et al, 2010), the change in miRNA expression will persist until at least 24 h thereafter, *i.e*. PID-10. Since there is a time lag between miRNA action and a change in the expression of the protein product of the final target gene(s), the physiological effect resulting from altered protein function could come about over several days thereafter. Another interesting finding from our profiling analyses is, among 43 potentially novel, unannotated miRNAs (classified as solexa sequences) several were observed to be strongly expressed in DRG and 10 showed significant dysregulation in expression in tumour-bearing as compared to sham-treated mice. It will be interesting in future studies to systematically elucidate the contributions of all annotated as well as novel miRNAs which were found to be dysregulated in conditions of bone metastatic pain.

Here, we chose miRNA-1a-3p as our prototypic miRNA for detailed mechanistic analyses owing to several reasons. One, although miR-1a-3p was characterized to be a non-neuronal miRNA with expression levels in the central nervous system 100- to 1000-fold lesser when compared to cardiac tissue (Mishima et al, 2007), later studies showed that miR-1a-3p is expressed in sensory neurons of the DRG in mice as well as humans (Bastian et al, 2011), which imparts it a particular translational significance; moreover, it has been well-characterized via *in situ* hybridization analysis to be localized to peptidergic nociceptors (Bastian et al, 2011). Secondly, mimicking miR-1a-3p in neuronal cultures attenuates neurite outgrowth (Bastian et al, 2011). Third, our results on regulation of miR-1a-3p expression in bone metastatic pain emerged to be intriguingly different to known findings in the context of neuropathic and inflammatory pain; whereas inflammatory pain as well as partial sciatic ligation are associated with a decrease in miR-1a-3p expression in the DRG and spinal cord, sciatic nerve axotomy induces a robust increase in miR-1a-3p expression in the DRG and a corresponding decrease in the spinal cord (Kusuda et al, 2011). Here, we observed that miRNA-1a-3p was highly upregulated in the DRG in bone metastatic pain. Despite insights into it's dysregulation in inflammatory and neuropathic states, the functions of miR-1a-3p in pain modulation had not been analysed so far. Our *in vivo* analyses revealed as miR-1a-3p in the DRG to be a functionally important positive modulator of bone metastatic pain, indicating a pronociceptive function for miR-1a-3p.

Based on these intriguing findings, we sought to identify genes regulated by miR-1a-3p in DRG neurons in an effort to work out mechanistic details. In doing so, we established and validated an approach to narrow down and select relevant genes from the very long lists of target genes, which typically emerge from *in silico* analyses. Thus, out of 62 mRNAs putative targets for miR-1a-3p predicted by at least 2 out of 14 algorithms applied, only 25 showed at least 50% up-regulation following miR-1a-3p inhibition in sensory neurons *in vivo*, thereby cautioning about the strength of interpretations that can be made from *in silico* analyses alone. Interestingly, except for *Hand2* and *Igf1*, almost all of genes that were functionally validated as miR-1a-3p targets in other systems were not found to be regulated by miR-1a-3p inhibition in sensory neurons, thereby highlighting the context-dependence of functionality of miRNA function and gene regulation. One of the most interesting aspects of this study was the identification of *Clcn3*, a chloride channel, as a functionally important gene target of miR-1a-3p in sensory neurons of the DRG. Apart from evidence for miRNA-1a-3p binding to the 3′UTR of *Clcn3* and regulation of it's translation, the finding that the expression of *Clcn3* expression is reciprocally regulated with respect to miR-1a-3p expression in the DRG following peripheral tumour induction established *Clcn3* as a miR-1a-3p target in sensory neurons. Furthermore, the phenotype of exaggerated tumour-mediated hyperalgesia evoked by specifically knocking *Clcn3* expression down in sensory neurons *in vivo* matched perfectly with attenuation of tumour-mediated hyperalgesia evoked by miR-1a-3p knockdown in the DRG. There are several ways via which *Clcn3*-mediated Cl^−^ flux could affect sensory neuron function. For example, it has been shown to be required for lysophosphatidic acid (LPA)-activated Cl^−^ current activity in myofibroblast differentiation (Yin et al, 2008), a molecular pathway which may be relevant to sensory neurons given that LPA is a highly potent and important modulator of sensory neuron function in pain disorders (Inoue et al, 2004; Ueda, 2008). Importantly, Clcn3 plays a role in both acidification and transmitter loading of GABAergic synaptic vesicles, which is very interesting in the light of the important role for GABAergic function in sensory terminals of nociceptors (Witschi et al, 2011). Moreover, in brain tissue, functional interactions between *Clcn3* and Ca^2+^/calmodulin-dependent protein kinase II have been reported (CamKII) (Cuddapah & Sontheimer, 2010; Huang et al, 2001), which could be relevant to the pain modulatory role of CamKII (Crown et al, 2012). Thus, the miR-1a-3p-*Clcn3* miRNA–mRNA regulation pair holds tremendous potential for modulating pain over multiple ways. A more detailed analysis of Clcn3 expression and sub-cellular distribution in the future could be very insightful; similarly, it will be interesting to study whether the role of Clcn3 observed here in the context of cancer pain also extends to the modulation of inflammatory or neuropathic pain states, given especially our observation that knocking down the basal expression of Clcn3 by itself significantly impacted on mechanical sensitivity in the absence of tumour growth.

Here, miR-34c-5p emerged as another key pronociceptive miRNA, which is induced in sensory neurons of the DRG in bone metastatic pain. Induction of this miRNA has been previously implicated in the hippocampus in the context of memory impairment and in the amygdala in the context of stress (Haramati et al, 2011; Zovoilis et al, 2011). Several notable pain modulatory genes are to be found amongst the *in silico* prediction list of miR-34c-5p target genes, including *Cacnb3* (the gene encoding calcium channel, voltage-dependent, beta 3 subunit), *Gabra3* and *Gabra1* (encoding GABA-A receptor, subunits alpha 3 and 1, respectively), *Scn2b* (sodium channel, voltage-gated, type II, beta), *Bdnf* (encoding brain derived neurotrophic factor), *Calca* (encoding calcitonin/calcitonin-related polypeptide, alpha), *Il6st* (encoding interleukin 6 signal transducer), *Ednrb* (endothelin receptor type B), amongst many others, which are known to significantly impact on pain (De Jongh et al, 2003; Enna & McCarson, 2006; Griswold et al, 1999; Knabl et al, 2008; Li et al, 2012; Murakami et al, 2007; Obata & Noguchi, 2006; Pedersen et al, 2007; Quarta et al, 2011; Waxman, 2010). Similarly, miR-370-3p, which was known in the context of angina pectoris and tumour suppression so far (Hoekstra et al, 2010; Zhang et al, 2012), emerged as a pro-nociceptive miRNA. Our *in silico* analyses revealed many interesting nociception related genes have binding sites for miR-370-3p, including gene encoding the purinergic receptor P2X, ligand-gated ion channel, 3 (*P2rx3*), opioid receptor, delta 1 (*Oprd1*), runt related transcription factor 1 (*runx1*), calcium channel, voltage-dependent, alpha 2/delta subunit 2 (*Cacna2d2*) among others, which have been characterized functionally in previous studies (Abrahamsen et al, 2008; Boroujerdi et al, 2011; Chen et al, 2006; Scherrer et al, 2010; Taylor & Garrido, 2008). Further detailed analyses will reveal which of these putative targets mediate the pronociceptive functions of miR-34c-5p and miR-370-3p in the context of cancer pain. Finally, two of the six dysregulated miRNAs analysed did not reveal a functional impact on tumour hypersensitivity. This is, however, not surprising since tumour–nerve interactions can encompass many other functional changes, *e.g*. cell death in the DRG, neuropathy, immune infiltration, etc., which were not studied here as readouts and represent interesting options for further detailed analyses.

Thus, the results of this study deliver valuable new insights into mechanisms of cancer pain. Furthermore, they open up very attractive therapeutic options since potential caveats associated with miRNAs and miRNA derivatives, *e.g*. ‘off-target’ effects, can be overwhelmed in this fast-developing field on the way towards the therapeutic development. Importantly, ncRNA-based diagnostics and therapeutics may have superior advantages by targeting multiple pain-associated genes simultaneously, and this study provides a preclinical basis in the context of cancer-associated pain.

## MATERIALS AND METHODS

### Animal model of tumour-evoked pain

All animal usage procedures were in accordance with ethical guidelines laid down by the local governing body (Regierungspräsidium Karlsruhe). All behavioural measurements were done in awake, unrestrained, age-matched adult (more than 2 months old) C3H/HeNCrl mice. The model of bone metastases-associated pain was implemented as described previously (Cain et al, 2001; Schweizerhof et al, 2009). Briefly, National Collection of Type Cultures (NCTC) clone 2472 fibrosarcoma cells (ATCC, Manassas, VA, USA) were cultured and injected into and around the calcaneus bone of wild type C3H/HeNCrl mice as described previously (Cain et al, 2001; Schweizerhof et al, 2009). An equal of saline was injected in the calcaneous bone in sham-treated mice.

### Measurement of mechanical sensitivity

Mice with tumour implantation or sham treatment were tested for paw withdrawal behaviour towards graded von Frey filaments (0.02, 0.07, 0.16, 0.4 and 1.0 g) applied to the plantar surface of the ipsilateral hind paw, as we have described in details previously (Schweizerhof et al, 2009). Prior to testing and tumour induction, mice were habituated to the experimental setup in at least two separate sessions. Mechanical sensitivity was represented as frequency of response, calculated as a percentage over five subsequent stimulations. Data are shown for some representative filaments, *e.g*. 0.07 or 0.02 g as well as in the form of area under the curve (AUC) calculated considering mean responses to all filament strengths from 0.02 to 1.0 g. Mechanical response threshold was calculated as von Frey filament strength required to achieve 50% (80% for Fig 3, panel B and Fig 6, panel F) withdrawal frequency. The experimentator was fully blinded to the identity of the treatment in all behavioural tests.

### RNA isolation

For gaining RNA in the context of microarray and qRT-PCR experiments, L3, L4 and L5 DRGs from tumour-bearing (described above) or control mice were pooled from four mice into one biological sample. All experiments were performed on triplicate biological samples. RNA isolation was carried out using mirVana™ miRNA Isolation Kit (Ambion, AM 1561) following manufacturer's instructions to enrich miRNA fraction and quality control was undertaken as described in details in Supporting Information Methods. Two hundred nanograms of total RNA from each biological sample was used as starting material for miRNA expression analysis.

### miRNA expression profiling and data analysis

Illumina Mouse Sentrix-6 beadchip arrays were used for miRNA expression arrays. Polyadenylation, 1st strand cDNA synthesis, hybridization of miRNA-specific oligos to the immobilized first strand cDNA, miRNA-specific second strand synthesis, universal PCR amplification and hybridization to the array were performed at the Genomic and Proteomics core facility, German Cancer Research Center, Heidelberg following in-house stringent quality control criteria. The array intensity data were imported into Beadstudio version 3.0 (Illumina), a software package that permits visualization and normalization of the data. We used the quantile normalization method (Chudin et al, 2006) for all the analyses reported here. To shortlist subset miRs regulated consistently in terms of biological and technical stability with more stringency for further analysis, we considered those miRNAs for which the distance between mean expression values between tumour-bearing and sham mice was at least five times greater than the sum of standard deviation of two groups. In analogical calculations, this value roughly equals to *p* < 0.001 (two tailed *t*-test assuming equal variances).

As the expression array was performed based on miRBase version 12 and owing to frequent dynamic changes in this online repository, all miRNA identifiers were updated according to current version of miRBase (version 19). However, to maintain the continuity of data representation and future reference purposes, sequences and accession numbers of 57 subset of significantly regulated miRNAs are provided in Supporting Information Table S1.

### PCR amplification of miRNAs and mRNAs

For the generation of miRNA-specific first strand cDNA, 20 ng of total RNA was reverse transcribed by miRNA specific RT primer using TaqMan® MicroRNA Reverse Transcription Kit (Applied Biosystems, 4366597) following manufacturer's instructions. Twenty nanograms of total RNA was used to prepare the cDNA using random primers from the High Capacity cDNA Reverse Transcription Kit (Applied Biosystems, 4368814) following manufacturer's instructions for mRNA amplification. Four microlitres of prepared cDNA was PCR amplified in each reaction using corresponding miRNA- or mRNA-specific primers using TaqMan® Universal Master Mix II, (Applied Biosystems, 4440040) following manufacturer's instructions on Chromo 4 detection system (BioRad, USA). The expression level of the target miRNA was normalized to expression of small nucleolar RNA 202 (sno202) and that of target mRNA was normalized to the expression of GAPDH. Each miRNA or mRNA was amplified in triplicates and *C*_t_ values were recorded. Fold change in the miRNA or mRNA expression in tumour bearing DRG samples over corresponding Sham samples in triplicate samples was calculated using *D*D*C*T method (Fu et al, 2006), which measures relative change in expression of a miRNA (or gene) from treatment to control compared to the reference small RNA (or gene). All miRNA and mRNA assays were purchased from Applied Biosystems and Assay IDs are as following: snoRNA202: 001232, miR-1: 002222, miR-34c-5p: 000428, miR-544-3p: 002550, miR-370-3p: 002275, miR-483*: 002560, miR-291b-5p: 002537; GAPDH: 4352932E; *Clcn3*: Mm01348786_m1.

### miRNA inhibitors and modified mimics

LNA based *in vivo* inhibitors for miR-1a-3p, 34c-5p and 544-3p and respective mismatch controls with several mismatches were custom ordered from Exiqon (Denmark). Sequences of inhibitor and mismatch inhibitors are given in the Supporting Information Table S2. miRIDIAN microRNA mimics for mmu-miR-370-3p (C-310619-07), miR-483* (C-310641-07) and miR-291b-5p (C-310666-03) were further custom-modified with 3′-cholesterol conjugation on passenger strand and 3′-FITC conjugation on guide strand to facilitate the *in vivo* uptake and facilitate visualization, respectively. Both inhibitors and mimics were synthesized from ribo-nucleotides and their functional efficacy *in vivo* was tested. These modified miRNA-mimics were custom ordered from Thermofischer Scientific Molecular biology (formerly Dharmacon, USA). Both mimics and inhibitors were purified via HPLC and lyophilized powder was reconstituted in 1× PBS at pH 7.4 at a concentration of 500 pmoles/µl, made into aliquots of working volumes and frozen at −20°C to avoid freeze–thaw cycles.

### Intrathecal application of miRNA/mRNA modulators *in vivo*

Mimic or inhibitor for each miRNA was applied intrathecally via subdurally implanted chronic catheters as we have described previously (Luo et al, 2012). On the day of application, inhibitors or mismatch inhibitors were mixed with 4 µl of i-Fect™ *in vivo* transfection reagent (Neuromics, Edina, MN) to a final concentration of 10 pmol/5 µl; injections were performed every 24 h over 5 consequent days starting on Day 4 after tumour implantation (PID-4 to PID-9). Lyophilized miRNA-mimics were reconstituted at a final concentration of 400 pmol/µl in 1× PBS at pH 7.4 and injected every 24 h over 7 consequent days from Day 0 to PID-7. Each injection of inhibitor or mimic was followed by a 5 µl saline flush. At the end of each experiment, catheter placement was evaluated via laminectomy and the data were only used from those mice, which showed correct catheter placement.

For *Clcn3* knock-down in DRGs, a set of three MISSION-predesigned siRNAs, which target the coding sequence of *Clcn3* mRNA (PDSIRNA5H-HA04065459, -HA04065461 and -HA04065463) or Universal siRNA negative control (8013156981) were purchased from Sigma–Aldrich Chemie GmbH (Germany). Intrathecal injections were performed every 24 h over 9 consequent days starting Day 0 until PID-8 after tumour implantation at a final concentration of 1.2 µg/µl per injection. The same methodology and scheme explained above for miRNA-inhibitors was used. For confirming siRNA-mediated knock-down of *Clcn3*, L3, L4, L5 DRGs were collected 48 h after a single injection of *Clcn3*- or control-siRNA and analysed for *Clcn3* expression by qRT-PCR.

**The paper explained**

**PROBLEM:**

Various forms of cancer are associated with debilitating pain. Approximately one-third of adults who are actively receiving treatment for cancer and two-thirds of those with advanced malignant disease experience pain. Various types of carcinomas and sarcomas metastasize to skeletal bones and cause spontaneous bone pain, hyperalgesia (exaggerated pain) and allodynia (pain in response to a normally innocuous stimulus), which is accompanied by bone degradation and remodelling of peripheral nerves. Cancer-mediated pain has both neuropathic and inflammatory components associated with it and it is difficult to treat the cancer-associated pain in a large number of clinical cases, particularly the neuropathic component involved. Current available therapeutics option or their application are severely limited owing to the widespread side effects. In order to develop novel, mechanism-based therapeutic strategies, it is imperative to delineate the cellular and molecular mechanisms underlying cancer-induced pain. miRNAs, a type of small non-coding RNAs in the genome, have recently been shown to be important for the mediation of various pathological conditions. A unique feature of miRNAs is that they act as ‘master switches’ in the genome by regulating many proteins at the same time, harbouring an enormous potential to be used as therapeutic targets. In the current study, we attempted to identify unique microRNA signatures and their mode of action associated with the cancer-mediated pain conditions by utilizing a clinically relevant model of metastatic bone-cancer pain in mice.

**RESULTS:**

We identified tumour-induced conditions are associated with a marked dysregulation of 57 miRNAs in peripheral sensory neurons, which relay the pain signals from the corresponding tumour-affected areas to the spinal cord and brain. We established effective protocols to target individual miRNAs specifically in the sensory neurons *in vivo* to test the importance of these deregulated miRNAs in the mediation of tumour-associated pain. Following our protocol, we identified three miRNAs namely miR-1a-3p, miR-34c-5p and miR-370-3p to be enhancing tumour-pain and miR-483-3p to counteract it. Our results indicate that reversing cancer-mediated overexpression of miR-1a-3p or miR-34c-5p or reversing tumour-mediated downregulation of miR-483-3p in the sensory neurons reduces tumour-associated pain. Furthermore, our detailed analyses identified the chloride channel 3 (*Clcn3*) as a target of miR-1a-3p in sensory neurons and functionally validated *Clcn3* as a novel pain modulator acting in the periphery.

**IMPACT:**

Our results of this study deliver valuable new insights into mechanisms of cancer pain and open up very attractive therapeutic options. This is particularly relevant since ncRNA-based diagnostics and therapeutics may have superior advantages by targeting multiple pain-associated genes simultaneously, and this study provides a preclinical basis in the context of cancer-associated pain. Our results underscore the importance of microRNA regulation in sensory neurons in the context of bone metastatic pain and systematically delineate the potential of ncRNAs as druggable targets for future treatment of cancer-associated pain.

### miRNA targets validation

Target prediction analysis was performed by comprehensively employing 14 state-of-art algorithms (see Supporting Information Table S3 and Supporting Information Methods for detailed list). Targets commonly predicted by at least 2 out of these 14 algorithms were analysed via quantitative RT-PCR using NanoString-nCounter™ based gene quantification method to validate microarray expression data. Probes specifically targeting desired gene of interest were ordered from the Nanostring Technologies (USA) who designed, synthesized and delivered the probes to the nCounter core facility at the Institute of human genetics, University clinic, Heidelberg, Germany. Two hundred nanograms of total RNA was used to analyse the target genes expression together with five housekeeping genes namely clathrin, heavy polypeptide (*Cltc*), glyceraldehyde-3-phosphate dehydrogenase (*Gapdh*), glucuronidase beta (*Gusb*), hypoxanthine guanine phosphoribosyl transferase (*Hprt*) and tubulin, beta 5 class I (*Tubb5*). Expression of target genes was analysed by comparing treated and control samples using normalized expression values.

### Data analysis and statistics

All data are expressed as mean ± standard error of the mean (SEM). Two-way repeated measures ANOVA followed by Bonferroni *post hoc* test was used to assess statistical significance in behavioural experiments. ANOVA followed by *post hoc* Fischer's test was used to assess statistical significance in QRT-PCR experiments. Student's *t*-test was used to assess statistical significance in reporter assays. Changes with *p* ≤ 0.05 were considered to be statistically significant.

## Supplementary material

As a service to our authors and readers, this journal provides supporting information supplied by the authors. Such materials are peer reviewed and may be re-organized for online delivery, but are not copy-edited or typeset. Technical support issues arising from supporting information (other than missing files) should be addressed to the authors.

**Review Process File**

**Figure S1. Lack of uptake of intrathecally administered miRNA-inhibitors in spinal cord neurons.**

**Figure S2. Functional validation of miRNAs upregulated in DRGs in tumour-bearing mice with respect to tumour-induced mechanical hypersensitivity.**

**Figure S3. Functional validation of miRNAs downregulated in DRGs in tumour-bearing mice with respect to tumour-induced mechanical hypersensitivity.**

**Figure S4. Representative images from the cryosections of lumbar DRG to confirm the lack of auto fluorescence or unspecific staining for images shown in** Fig 5 **panel E.**

**Figure S5. Effect of siRNA-mediated *Clcn3* knock-down in the DRGs on the basal mechanical sensitivity.**

**Table S1.** Complete data over miRNAs, which were found to be significantly upregulated or downregulated via microarray analysis in ipsilateral lumbar DRG on Day 8 following induction of tumour growth in the calcaneous bone of the heel in mice as compared to sham surgery.

**Table S2.** Sequences of LNA-based miRNA-inhibitors, which were custom designed for miRNA inhibition in the DRGs *in vivo*.

**Table S3.** Summary of top 10 genes, which represent predictions as targets of miR-1a-3p via *in silico* analysis using 14 different algorithms.
