# Supplementary material for: Genome-wide identification and functional analyses of microRNA signatures associated with cancer pain
Source: EMBO Mol Med. 2013 Oct 18;5(11):1740–58. doi: 10.1002/emmm.201302797 (PMC3840489; doi:10.1002/emmm.201302797)
Supplement: Supplementary file 1 [file emmm0005-1740-SD1.pdf]

Genome-wide identification and functional analyses of microRNA signatures associated with cancer pain

Kiran Kumar Bali, Deepitha Selvaraj, Venkta P. Satagopam, Jianning Lu, Reinhard Schneider and Rohini Kuner

Corresponding author: Rohini Kuner, Institute of Pharmacology, Heidelberg University

Review timeline:

Submission date:	23 March 2013
Editorial Decision:	17 April 2013
Revision received:	25 July 2013
Editorial Decision:	14 August 2013
Revision received:	22 August 2013
Accepted:	27 August 2013

Transaction Report:

(Note: With the exception of the correction of typographical or spelling errors that could be a source of ambiguity, letters and reports are not edited. The original formatting of letters and referee reports may not be reflected in this compilation.)

Editor: Roberto Buccione

1st Editorial Decision 17 April 2013

Thank you for the submission of your manuscript to EMBO Molecular Medicine. We have now heard back from the three Reviewers whom we asked to evaluate your manuscript.

You will see that all three Reviewers are generally supportive of you work although Reviewer 1, in particular, expresses some concerns that prevent us from considering publication at this time.

Reviewer 1, while generally quite positive, points to a number of experimental issues that require your action. S/he is especially concerned that key experiments lack sufficient controls and that imaging and statistical analysis need to be significantly improved. I will not dwell into much detail, as the items are detailed, self-explanatory and well taken.

Reviewer 2 has a few concerns that also require your attention.

Considered all the above, while publication of the paper cannot be considered at this stage, we would be pleased to consider revised submission, with the understanding that the Reviewers' concerns must be fully addressed, with additional experimental data where appropriate and that acceptance of the manuscript will entail a second round of review.

Please note that it is EMBO Molecular Medicine policy to allow a single round of revision only and that, therefore, acceptance or rejection of the manuscript will depend on the completeness of your responses included in the next, final version of the manuscript.

As you know, EMBO Molecular Medicine has a "scooping protection" policy, whereby similar

findings that are published by others during review or revision are not a criterion for rejection. However, I do ask you to get in touch with us after three months if you have not completed your revision, to update us on the status. Please also contact us as soon as possible if similar work is published elsewhere.

I look forward to seeing a revised form of your manuscript as soon as possible.

***** Reviewer's comments *****

Referee #1 (General Remarks):

The study touches upon an extremely important and physiologically relevant topic of pain research, specifically, molecular mechanisms underlying the elevation of pain during cancer conditions. There could be myriad mechanisms underlying pain/hyperalgesia due to the plethora of cancer conditions and activated pathways. It is agreed that mechanisms underlying chronic pain under cancer conditions are a current, relevant topic. In this respect, the authors correctly noted that "various chronic diseases are associated with unique signatures of expression of microRNAs (miRNAs), which reveal deep insights into disease pathology". Selected model for cancer conditions - metastatic bone-cancer pain in mice - is indeed clinically relevant. Selected approaches are also appropriate. Altogether, I have no concern regarding rationale, approaches, and a majority of interpretations. However, this reviewer would like to see more controls (outlined below), micro-fluorescent pictures that illustrate whether oligos and mimic miRNA migrate into spinal cord cells, re-evaluation of statistical data and higher quality anatomical pictures.

1. Abstract: "tumor-induced hypersensitivity is associated with a marked dysregulation of 56 miRNAs". I recommend writing: "tumor-induced conditions....", because hypersensitivity was validated for 6 miRNA, but not all of 56 miRNAs.
2. Why was regulation of miRNA studied in sensory neurons? Rationale on selection of DRG over spinal cord cells would be helpful in the "introduction" section.
3. What does "remodeling" of sensory neurons mean? There are several possible types (peripheral sprouting, central terminal sprouting and so on) for this remodeling. This should be expanded and specified.
4. Page 4: Hind paw is innervated by L4-L6, but not L3 and L4 ganglia. Otherwise, citation should be included.
5. Fig 1E. Letter "E" is not on the cartoon. Probably, D is E; and D is unmarked PID-8 data.
6. Are inhibitor oligos and mimic miRNAs synthesized from ribo-nucleotides? This is a critical issue, since some believe that double-stranded DNA cannot mimic dsRNA. This point needs to be specified in the text or in the methods section.
7. For Fig 2B what percentage of DRG neurons has up-taken oligos? I have counted 10-15% from provided picture (2B; bottom right panel). Are there some other images? Presented images are not high quality (too blurry). The percentage of neurons that have up-taken oligos should be reflected in the text. Importantly, what about spinal cord cells? Have they up-taken oligos? This has to be examined.
8. Fig 2D (Right side columns). Is statistic only $p<0.05$? Error bars and mean difference looks more substantial.
9. The authors stated: "Based on previous observations that intrathecally-delivered fluorescence probe-tagged short-oligonucleotides can be detected in the DRGs only up to 24 hours (h) following injection". However, injection was stopped at PID-9 and effect persisted 5 more days (Fig 3). Are there explanations for this?
10. Fig 3. Have you analyzed threshold (g) for withdrawal to mechanical stimuli? Threshold analysis/measurement is more reflect mechanical allodynia, which is especially clinically relevant.
11. For Fig 3 and 4: Two-way ANOVA must be used. Post-hoc test has to be Bonferroni. One-way ANOVA is not acceptable for this type of data.
12. Fig 5E (Clcn3): Labeling quality for Clcn3 is not convincing. Do you have control for auto-fluorescence for secondary antibodies?
13. Fig 3, 4, 6E should contain graph for hypersensitivity, when only vehicle is injected, and conventional bone concern pain for Fig 4. This is a very important data, since effect of gross mismatch oligos and mimics cannot be evaluated. Thus, mismatch oligos and mimics could have statistically significant effects by themselves, but not compare to corresponding experimental oligos

and mimics.
Overall, this is excellent work. However, above outlined additional experiments and revisions need to be performed to substantially increase solidity of findings. Thus, there are ongoing, vigorous debates on whether sensory neuronal or spinal cord miRNAs is more important for chronic pain conditions.

Referee #2 (Comments on Novelty/Model System):

This paper includes high quality and well controlled approaches for examining gene regulation in mice with cancer pain. The results are novel since this has not been done with this tumor model, and the studies of Clcn3 gene is particulalrly nove and exciting because of the potential role in modulating neuronal excitability in nociceptors. The medical impact is potentially high since identifying changes in gene expressionthat contribute to cancer pain may lead to novel treatment approaches. The model of cancer pain is adequate and has been used in several earlier studies of pharacological and electrophysiological studies of cancer pain.

Referee #2 (General Remarks):

This is a well written and interesting paper with nove and important findings. Identifying genes thatare dysregulated and correlate with the development of cancer pain is new and can potentially lead to new targets fro treating cancer-related pain. The studies of the Clcn3 gene are particularly exciting. In general, the studies are well designed, the data are presented clearly, and the discussion is consistent with the results. The results are novel and important to the field. There are only a few minor concerns that should be addressed.

1. "E" in Figure 1 needs to be labeled.
2. Are any of the genes that were regulated by tumor growth also regulated by other pain states, such as inflammation of nerve injury? Both of these conditions occur with tumor growth, so can it be know whether the results of the present study are associated with inflammation, nerve injury, or specific changes resulting from substances released by the tumor cells?

3. Did Clcn3 siRNA produce hyperalgesia in control mice?

Referee #3 (Comments on Novelty/Model System):

This is a very strong paper, with a novel insight into pain mechanisms and the role of microRNAs in cancer pain. The potential medical impact from this is high, and could be extended to other pain syndromes.

Referee #3 (General Remarks):

This is an excellent paper providing novel insights into cancer pain mechanisms. I wonder if any of these insights into particular microRNAs and chloride or other channel targets can be extended to additional pain syndromes? I find this paper compelling and of a very high quality. It would be interesting to see microarray data in the cancer pain model, and an analysis of the role of Clcn3 in other pain syndromes.

1st Revision - authors' response 25 July 2013

The authors would like to thank all reviewers for their insightful comments and constructive suggestions. We have now performed new experiments and revised the manuscript along the lines suggested by the reviewers. Please find a detailed list of point-point responses below. Changes in the manuscript text are marked in a bold font.

Referee #1 (General Remarks):

The study touches upon an extremely important and physiologically relevant topic of pain research, specifically, molecular mechanisms underlying the elevation of pain during cancer conditions. There could be myriad mechanisms underlying pain/hyperalgesia due to the plethora of cancer conditions and activated pathways. It is agreed that mechanisms underlying chronic pain under cancer conditions are a current, relevant topic. In this respect, the authors correctly noted that "various chronic diseases are associated with unique signatures of expression of microRNAs (miRNAs), which reveal deep insights into disease pathology". Selected model for cancer conditions - metastatic bone-cancer pain in mice - is indeed clinically relevant. Selected approaches are also appropriate. Altogether, I have no concern regarding rationale, approaches, and a majority of interpretations. However, this reviewer would like to see more controls (outlined below), micro-fluorescent pictures that illustrate whether oligos and mimic miRNA migrate into spinal cord cells, re-evaluation of statistical data and higher quality anatomical pictures.

1. Abstract: "tumor-induced hypersensitivity is associated with a marked dysregulation of 56 miRNAs". I recommend writing: "tumor-induced conditions....", because hypersensitivity was validated for 6 miRNA, but not all of 56 miRNAs.

Response: We agree with the reviewer and have changed the sentence accordingly in the abstract.

2. Why was regulation of miRNA studied in sensory neurons? Rationale on selection of DRG over spinal cord cells would be helpful in the "introduction" section.

Response: We agree with the reviewer. Both peripheral as well as spinal contributions are of importance in understanding and eventually treating cancer pain (Mantyh, 2004). A key interest of the authors is to address plasticity mechanisms at the interface between tumor cells and nociceptive pathway; thereby, mechanisms operational in sensory neurons are of prime interest. This is supported by numerous studies which have demonstrated changes in the structure as well as the function of sensory neurons in cancer pain states, which are attributed to effects of tumor growth and tumor-associated mediators (Cain et al, 2001; Constantin et al, 2008; Mantyh, 2004; Schweizerhof et al, 2009). Another reason to target sensory neurons is that the behavioral assays we employ essentially permit the analysis of primary hyperalgesia, i.e. changes in nociceptive sensitivity at the site of tumor growth. Therefore, it makes complete sense to study changes in miRNA profiles in DRG neurons.

These aspects are described on page 3, lines 24-32 of the revised manuscript.

3. What does "remodeling" of sensory neurons mean? There are several possible types (peripheral sprouting, central terminal sprouting and so on) for this remodeling. This should be expanded and specified.

Response: Here, the term ‘remodelling’ was meant in a structural sense, i.e. changes such as hypertrophy and sprouting of sensory nerves, which have been reported in the model of cancer pain utilized here. This has now been specified in the text on page 5, lines 11-12 of the revised manuscript.

4. Page 4: Hind paw is innervated by L4-L6, but not L3 and L4 ganglia. Otherwise, citation should be included.

Response: The reviewer is right in that in rats, the hindpaw is predominantly innervated by L4-L6. However, it has been demonstrated in mice that the hindpaw is predominantly innervated by L3 & L4 (Rigaud et al, 2008). It is a standard practice in most labs to study L3 & L4 in studies pertaining to mouse and this has been reflected in several published studies from our laboratory as well (e.g. Schweizerhof et al. Nature Medicine, 2009; Gangadharan et al. J. Clin. Investigation. 2011, Luo et al. Plos Biology, 2012, amongst others). We have now clarified this in the text and cited an appropriate reference on page 5, lines 15-16.

5. Fig 1E. Letter "E" is not on the cartoon. Probably, D is E; and D is unmarked PID-8 data.

Response: We thank the reviewer for pointing this out. Fig.1 has now been revised accordingly.

6. Are inhibitor oligos and mimic miRNAs synthesized from ribo-nucleotides? This is a critical issue, since some believe that double-stranded DNA cannot mimic dsRNA. This point needs to be specified in the text or in the methods section.

Response: Both the inhibitors and mimics were synthesized from ribo-nucleotides and were effective in inhibiting or mimicking the expression of miRNAs, respectively, as shown in Fig. 2 panels C and D. This point has now been clarified in the 'Methods' section on page 20, lines 15-16.

7. For Fig 2B what percentage of DRG neurons has up-taken oligos? I have counted 10-15% from provided picture (2B; bottom right panel). Are there some other images? Presented images are not high quality (too blurry). The percentage of neurons that have up-taken oligos should be reflected in the text. Importantly, what about spinal cord cells? Have they up-taken oligos? This has to be examined.

Response: We thank the reviewer for these valuable suggestions. We now performed new experiments to evaluate the extent of intrathecally administered miRNA inhibitor and mimic uptake into DRGs and spinal cord. Because a single molecule of the fluorescent moiety, FITC, is conjugated to each molecule of the oligo, one does not see very bright fluorescent signals. However, when we performed carefully controlled confocal imaging, we observed fluorescently-marked molecules in DRG neurons within 24 hrs of their intrathecal application, but not in the spinal cord. This is not surprising, since intrathecally delivered agents are known to typically reach DRGs more efficiently than spinal cord.

Quantitative analyses are difficult, but we estimate that about 73% of DRG neurons take up the labeled inhibitors.

We have now included higher resolution images of DRG neurons in Fig. 2B and of the spinal dorsal horn in Suppl. Fig. 1. This is now clarified on page 7, lines 17-23 of the revised manuscript.

8. Fig 2D (Right side columns). Is statistic only $p<0.05$? Error bars and mean difference looks more substantial.

Response: The actual statistical P value for the data the reviewer is referring to in Fig. 2D was 0.00. However, throughout the manuscript, we have considered P vales less than 0.05 to be statistically significant. Therefore, to stay consistent with figures, we have would like to maintain this way of representation.

9. The authors stated: "Based on previous observations that intrathecally-delivered fluorescence probe-tagged short-oligonucleotides can be detected in the DRGs only up to 24 hours (h) following injection". However, injection was stopped at PID-9 and effect persisted 5 more days (Fig 3). Are there explanations for this?

Response: We agree with the reviewer that this is indeed an intriguing point. Our interpretation of the data is that even though injection of miRNA modulators was stopped at PID-9, the change in miRNA expression will be come about (or persist) until at least 24 h thereafter, i.e. PID-10. Since there is a time lag between miRNA action and a change in the expression of the protein product of the final target gene(s), the physiological effect resulting from altered protein function could come about over several days thereafter. Indeed we have observed in a previous study (Schweizerhof et al. 2009), that inhibiting the function of the GM-CSF receptor could alter cancer-associated mechanical hypersensitivity for several days after the last injection of the reagent.

10. Fig 3. Have you analyzed threshold (g) for withdrawal to mechanical stimuli? Threshold analysis/measurement is more reflect mechanical allodynia, which is especially clinically relevant.

Response: We thank the reviewer for this insightful suggestion. We now included the analysis performed taking 50% response frequency as a parameter to calculate the mechanical threshold to von Frey stimulation in the revised results. Accordingly, we have replaced the Area Under Curve (AUC) panels from Fig. 3, 4 and 6 with threshold analyses and presented the AUC data in revised supplementary fig.2

11. For Fig 3 and 4: Two-way ANOVA must be used. Post-hoc test has to be Bonferroni. One-way ANOVA is not acceptable for this type of data.

Response: We thank the reviewer for this constructive suggestion. We have performed Two-way ANOVA with Bonferroni post-hoc test for the relevant data and did not observe any major deviations from our previous results and inferences. The legends of Fig. 3, 4, 6, the methods and results sections have now been revised accordingly.

12. Fig 5E (Clcn3): Labeling quality for Clcn3 is not convincing. Do you have control for auto-fluorescence for secondary antibodies?

Response: Yes, we performed a control for autofluorescence with secondary antibodies alone and observed a lack of staining (shown in new Supp. Fig. 4). Unfortunately, the current availability of antibodies against Clcn3 is very limited. We have now acknowledged in the discussion section that a more detailed future analysis of Clcn3 expression and function would be potentially very insightful. These aspects are mentioned on page 11, line 36 to page 12 line1 of the results and discussed on page 16, lines 22-28 of the discussion.

13. Fig 3, 4, 6E should contain graph for hypersensitivity, when only vehicle is injected, and conventional bone concern pain for Fig 4. This is a very important data, since effect of gross mismatch oligos and mimics cannot be evaluated. Thus, mismatch oligos and mimics could have statistically significant effects by themselves, but not compare to corresponding experimental oligos and mimics.

Response: We agree with the reviewer. The revised figures now represent the vehicle only group.

Overall, this is excellent work. However, above outlined additional experiments and revisions need to be performed to substantially increase solidity of findings. Thus, there are ongoing, vigorous debates on whether sensory neuronal or spinal cord miRNAs is more important for chronic pain conditions.

Referee #2 (Comments on Novelty/Model System):

This paper includes high quality and well controlled approaches for examining gene regulation in mice with cancer pain. The results are novel since this has not been done with this tumor model, and the studies of Clcn3 gene is particulalrly nove and exciting because of the potential role in modulating neuronal excitability in nociceptors. The medical impact is potentially high since identifying changes in gene expressionthat contribute to cancer pain may lead to novel treatment approaches. The model of cancer pain is adequate and has been used in several earlier studies of pharacological and electrophysiological studies of cancer pain.

Referee #2 (General Remarks):

This is a well written and interesting paper with nove and important findings. Identifying genes thatare dysregulated and correlate with the development of cancer pain is new and can potentially lead to new targets fro treating cancer-related pain. The studies of the Clcn3 gene are particularly exciting. In general, the studies are well designed, the data are presented clearly, and the discussion is consistent with the results. The results are novel and important to the field. There are only a few minor concerns that should be addressed.

1. "E" in Figure 1 needs to be labeled.

Response: We thank the reviewer for noticing this error, which has now been corrected in revised Fig. 1.

2. Are any of the genes that were regulated by tumor growth also regulated by other pain states, such as inflammation of nerve injury? Both of these conditions occur with tumor growth, so can it

be know whether the results of the present study are associated with inflammation, nerve injury, or specific changes resulting from substances released by the tumor cells?

Response: Since the current study reports profiling expression of miRNAs, not genes (mRNAs), we assume that the reviewer's question pertains to commonalities in miRNA expression & regulation across models for cancer pain and neuropathic and inflammatory pain. Indeed, there are not many studies which have comprehensively profiled miRNAs expression in the DRG at different time points in models of neuropathic and inflammatory pain. Most studies deal with candidate miRNAs which were analysed on an individual basis. Furthermore, there are several reports of spinal expression of miRNAs, which, however, cannot be compared with the present results derived from DRG.

Upon comparing our results with the small volume of published literature on DRG, we found very few commonalities. For example, we have already discussed the highly differential course of modulation of miR-1a-3p in the DRG across diverse chronic pain models on page 15, lines 12-22 (now made bold in revised manuscript). As described, the nature of modulation of miR-1a-3p in the DRG appears to differ even across different neuropathic pain models! Thus, miRNA1 is downregulated in the DRG upon paw inflammation or partial nerve injury (Kusuda et al, 2011), and paradoxically reported to be upregulated after sciatic nerve axotomy model (Kusuda et al, 2011), similar to our observation of an induction in a tumor pain model. We did not find any other commonalities pertaining to miRNA regulation in the DRG across pain models.

Thus, there appears to be very little overlap between regulation of miRNAs across models of diverse types of pathological pain. However, we believe that it is too premature to comment on this because comprehensive studies spanning a large set of time points and tissues will be required before meaningful comparisons can be made across diverse pain models. It is indeed our interest to compare miRNA signatures across diverse pain states, but this is a topic for future analyses and goes beyond the scope of the current manuscript, which was designed to focus on cancer pain and is, by itself, very long and detailed.

3. Did Clcn3 siRNA produce hyperalgesia in control mice?

Response: We thank the reviewer for this important question. Indeed, we have now performed the interesting experiment suggested by the reviewer and observed that *Clcn3* knock-down in DRGs led to a small, but statistically significant, increase in basal mechanical sensitivity and a significant decrease in the basal mechanical nociceptive threshold. These new data have been presented in Suppl. Fig. 5, described on page 13, lines 11-21 of the results and discussed on page 16, lines 26-28.

Referee #3 (Comments on Novelty/Model System):

This is a very strong paper, with a novel insight into pain mechanisms and the role of microRNAs in cancer pain. The potential medical impact from this is high, and could be extended to other pain syndromes.

Referee #3 (General Remarks):

This is an excellent paper providing novel insights into cancer pain mechanisms. I wonder if any of these insights into particular microRNAs and chloride or other channel targets can be extended to additional pain syndromes? I find this paper compelling and of a very high quality. It would be interesting to see microarray data in the cancer pain model, and an analysis of the role of Clcn3 in other pain syndromes.

Response: We thank the reviewer for the insightful comments. The current study was designed to address potential dysregulation of microRNAs in sensory neurons in cancer pain states via microarray analysis. The question about whether miRNAs which emerged to be important in cancer pain modulation also come into play in other pain states is indeed very intriguing. As reported on page 15, lines 12-22 (now made bold in revised manuscript), we have already discussed the highly differential course of modulation of miR-1a-3p in the DRG across diverse models of inflammatory,

neuropathic and cancer pain. However, beyond this, we did not find commonalities across diverse pain models. The reasons for this and the danger of making premature inferences are discussed in details on the previous page in response to question 1 from review # 2 (please see page 6).

Similarly, there is no literature so far on the role of Clcn3 in neuropathic or inflammatory states. While doing such an analysis, it will be important to study potential dysregulation of Clcn3 expression as well as functional modulation at various time points over the course of these disorders. This is particularly important because the same gene can undergo various cycles of changes in expression (at times, bidirectional modulation) over the various phases in pain models (e.g. Simonetti et al. Neuron, 2013). Therefore, a highly comprehensive analysis will be required to enable meaningful comparisons. Therefore, such experiments, albeit extremely intriguing, are beyond the scope of the present study and will be the focus of future studies. This is now discussed pn page 16, lines 22-28.

References:

Cain DM, Wacnik PW, Turner M, Wendelschafer-Crabb G, Kennedy WR, Wilcox GL, Simone DA (2001) Functional interactions between tumor and peripheral nerve: changes in excitability and morphology of primary afferent fibers in a murine model of cancer pain. *J Neurosci* 21(23): 9367-9376

Constantin CE, Mair N, Sailer CA, Andratsch M, Xu ZZ, Blumer MJ, Scherbakov N, Davis JB, Bluethmann H, Ji RR, Kress M (2008) Endogenous tumor necrosis factor alpha (TNFalpha) requires TNF receptor type 2 to generate heat hyperalgesia in a mouse cancer model. *J Neurosci* 28(19): 5072-5081

Kusuda R, Cadetti F, Ravanelli MI, Sousa TA, Zanon S, De Lucca FL, Lucas G (2011

) Differential expression of microRNAs in mouse pain models. *Mol Pain* 7: 17

Mantyh PW (2004) A mechanism-based understanding of bone cancer pain. *Novartis Found Symp* 261: 194-214; discussion 214-199, 256-161

Rigaud M, Gemes G, Barabas ME, Chernoff DI, Abram SE, Stucky CL, Hogan QH (2008) Species and strain differences in rodent sciatic nerve anatomy: implications for studies of neuropathic pain. *Pain* 136(1-2): 188-201

Schweizerhof M, Stosser S, Kurejova M, Njoo C, Gangadharan V, Agarwal N, Schmelz M, Bali KK, Michalski CW, Brugger S, Dickenson A, Simone DA, Kuner R (2009) Hematopoietic colony-stimulating factors mediate tumor-nerve interactions and bone cancer pain. *Nat Med* 15(7): 802-807

2nd Editorial Decision 14 August 2013

Thank you for the submission of your revised manuscript to EMBO Molecular Medicine. We have now received the enclosed reports from the two Reviewers that were asked to re-assess it. As you will see below, the reviewers are now globally supportive pending some final requests, and I am pleased to inform you that we will be able to accept your manuscript pending the following final amendments which will be dealt with editorially:

1) Reviewer 1 suggests that items 8 and 9 of your rebuttal to his/her comments be incorporated in the text. I would suggest that you do so for 9. As for item 8, please see my point 3 further below.

2) Reviewer 2 would like you to discuss a few additional points. I agree, although you should do so

concisely. S/he also notes that there is an inconsistency in the description for figure 1.

3) As per our Author Guidelines, the description of all reported data that includes statistical testing must state the name of the statistical test used to generate error bars and P values, the number (n) of independent experiments underlying each data point (not replicate measures of one sample), and the actual P value for each test (not merely 'significant' or 'P < 0.05').

Please submit your revised manuscript within two weeks. I look forward to seeing a revised form of your manuscript as soon as possible.

I look forward to reading a new revised version of your manuscript as soon as possible and in any case, within two weeks.

***** Reviewer's comments *****

Referee #1 (General Remarks):

This is a terrific and comprehensive revision of the previous manuscript. I am satisfied with the new data and explanations for previous questions. Answers to questions 8 and 9 could be included into the text (but this is optional).
Overall, the study is really well done and good luck with future research.

Referee #2 (Comments on Novelty/Model System):

The authors use an established model of cancer pain to investigate underlying mechanisms. The approach of studying miRNA to the application of cancer pain is novel and the data are well presented, interesting, new, and have clinical relevance.

Referee #2 (General Remarks):

This is a well-designed study with novel and important resuts. The data re presentled clearly and there is an impressive mix of genetics, molecular biology and behavior to address the role of certain miRNA to the development of cancer pain. There are only a couple of suggestions for improvement.

1. It would be interesting if similar changes in miRNA would be seen if the bone was not involved. Subcutaneous injection into the hind paw of the same sarcoma cells produces hyperalgesia, although the magnitude is less than with bone destruction. Are there any data on changes in miRNA after subcutaneous injection of the cancer cells that avoid the bone? How much of the changes observed in the present study have to do with bone pain vs. cancer-related pain? Thsi can be discussed briefly.

2. Page 5. Figure 1B is described as showing stimulus-response frequency over graded von Frey filaments, but the actual figure shows mechanical response threshold over time.

2nd Revision - authors' response 22 August 2013

Responses to reviewers by Bali et. al.

The authors would like to thank the reviewers for their insightful comments and suggestions. We have now revised the manuscript along the lines suggested by the reviewers. Please find a detailed list of point-point responses below. Changes in the manuscript text are marked in a bold font.

Referee #1 (General Remarks):

This is a terrific and comprehensive revision of the previous manuscript. I am satisfied with the new data and explanations for previous questions. Answers to questions 8 and 9 could be included into the text (but this is optional). Overall, the study is really well done and good luck with future research.

Response: We thank the reviewer for the suggestion. We have now incorporated the point about the miRNA-mediated temporal regulation of target genes in the revised manuscript (point 8) in the discussion section (page 14, line 36 to page 15 line 6). For the point 9 about the statistical significance of the presented data, we have now presented precise significance P values for each data point in all the figures and changed the legends of all figures and supplementary figures accordingly in the revised manuscript.

Response:

Referee #2 (Comments on Novelty/Model System):

The authors use an established model of cancer pain to investigate underlying mechanisms. The approach of studying miRNA to the application of cancer pain is novel and the data are well presented, interesting, new, and have clinical relevance.

Referee #2 (General Remarks):

This is a well-designed study with novel and important resuts. The data representled clearly and there is an impressive mix of genetics, molecular biology and behavior to

address the role of certain miRNA to the development of cancer pain. There are only a couple of suggestions for improvement.

1. It would be interesting if similar changes in miRNA would be seen if the bone was not involved. Subcutaneous injection into the hind paw of the same sarcoma cells produces hyperalgesia, although the magnitude is less than with bone destruction. Are there any data on changes in miRNA after subcutaneous injection of the cancer cells that avoid the bone? How much of the changes observed in the present study have to do with bone pain vs. cancer-related pain? Thsi can be discussed briefly.

Response: We thank the reviewer for this suggestion. We have now included this point in the discussion section (page 14, lines 14-18) in the revised manuscript.

2. Page 5. Figure 1B is described as showing stimulus-response frequency over graded von Frey filaments, but the actual figure shows mechanical response threshold over time.

Response: We thank the reviewer for pointing this out and apologize for the mistake. We have now corrected this in the revised manuscript (page 5 lines 24-29).
