# Supplementary material for: Genome-wide identification and functional analyses of microRNA signatures associated with cancer pain
Source: EMBO Mol Med. 2013 Oct 18;5(11):1740–58. doi: 10.1002/emmm.201302797 (PMC3840489; doi:10.1002/emmm.201302797)
Supplement: Supplementary file 2 [file emmm0005-1740-SD2.pdf]

Genome-wide identification and functional analyses of microRNA signatures associated with cancer pain

Kiran Kumar Bali[1,2], Deepitha Selvaraj[1,2], Venkta P. Satagopam[3,4], Jianning Lu[1,2], Reinhard Schneider[3,4], Rohini Kuner[1,2]

1. Institute for Pharmacology, Medical Faculty Heidelberg, Heidelberg University, Im Neuenheimer Feld 366, D-69120 Heidelberg, Germany
2. Molecular Medicine Partnership Unit with European Molecular Biology Laboratory, Meyerhofstrasse 1, D-69117 Heidelberg, Germany
3. Luxembourg Centre for Systems Biomedicine (LCSB), University of Luxembourg, Campus Belval, House of Biomedinice, 7 avenue des Hauts-Fourneaux, L-4362 Esch-sur-Alzette, Luxembourg
4. European Molecular Biology Laboratory, Meyerhofstrasse 1, D-69117 Heidelberg, Germany

Supplementary materials:

Table of Contents:

Supplementary methods:

- miRNA isolation and quality control ……………………………………………………..3
- Immunohistochemistry on DRG sections……………………………………………..3
- Cloning of *Clcn3* 3'UTR into reporter vector…………………………………………3
- HEK cell culture and luciferase assay………………………………………………..4
- Prediction of miRNA-1a-3p targets……………………………………………………4

Supplementary Figures:

- Suppl. Fig.1. Lack of uptake of intrathecally-administered miRNA-inhibitors in spinal cord neurons……………………………………………………………………...5
- Suppl. Fig.2. Functional validation of miRNAs upregulated in DRGs in tumor-bearing mice with respect to tumor-induced mechanical hypersensitivity…………6
- Suppl. Fig.3. Functional validation of miRNAs downregulated in DRGs in tumor-bearing mice with respect to tumor-induced mechanical hypersensitivity…………8

- Suppl. Fig.4. Representative images from the cryosections of lumbar DRG to confirm the lack of auto fluorescence or unspecific staining for images shown in Fig 5 panel E…………………………………………………………………….…9
- Suppl. Fig.5. Effect of siRNA-mediated *Clcn3* knock-down in the DRGs on the basal mechanical sensitivity…………………………………………………………10

Supplementary Tables:

- **Supplementary Table 1:** Complete data over miRNAs which were found to be significantly upregulated or downregulated via microarray analysis in ipsilateral lumbar DRG on day 8 following induction of tumor growth in the calcaneous bone of the heel in mice as compared to sham surgery……………………………..……12
- **Supplementary Table 2: Sequences of** LNA-based miRNA-inhibitors which were custom designed for miRNA inhibition in the DRGs *in vivo*…………………14
- **Supplementary Table 3:** Summary of top 10 genes which represent predictions as targets of miR-1a-3p via *in silico* analysis using 14 different algorithms…..…15

References………………………………………………………………………………………17

Supplementary methods:

miRNA isolation and quality control: Mice were killed using CO_2, spinal column isolated, rinsed in cold 1X PBS and Lumbar level 3, 4 and 5 DRGs were quickly isolated into a micro centrifuge tube and flash frozen in liquid nitrogen until RNA isolation is performed. Total RNA was isolated using mirVana™ miRNA Isolation Kit (Ambion, AM 1561) following manufacturer's instructions to enrich miRNA fraction by adding 1.25 times of absolute ethanol to the upper phase isolated from DRG lysate + Chloroform:Phenol mixture. RNA was dissolved in nuclease free water. Purification steps were performed using RNAse-free DNAse kit (Qiagen, 79254) following manufacturer's instructions. RNA concentration was determined using the NanoDrop spectrophotometer (NanoDrop Technologies, Wilmington, DE) and the quality of total RNA was checked by gel analysis using the total RNA Nanochip assay on an Agilent 2100 Bioanalyzer (Agilent Technologies GmbH, Waldbronn, Germany). Only samples with RNA index values greater than 7 were selected for microRNA profiling. 200ng of total RNA from each biological sample was used as starting material for miRNA expression analysis.

Immunohistochemistry on DRG sections: Mice were transcardially perfused with cold phosphate-buffered saline (PBS) and 4% cold paraformaldehyde (PFA) and L3-L4 DRGs were extracted, cryopreserved in sucrose and cryosectioned at 16 µm thickness. Cryotome sections of wild-type mouse DRGs were stained with anti-Clcn3 antibody (1:500; Sigma, AV35504) using standard protocols for immunoflorescence staining (Schweizerhof et al, 2009). Anti-CGRP antibody (1:300; 24112, Immunostar, Germany) and biotinylated-Isolectin B4 (1:200; B-1205, Vector, Burlingame, CA, USA) were used to label peptidergic and non-peptidergic nociceptive neurons, respectively, using standard protocols and analyzed using a confocal laser-scanning microscope (Leica TCS SP3 AOBS). Hoechst/DAPI nuclear staining was used to visualize the nucleus.

Cloning of *Clcn3* 3'UTR into reporter vector: A primer set (5' AGA CCC CGC TTC AAT AAT GTT C 3' and 5' ACA CGC TCG CCC ATT TTC ATC AT 3') was designed to amplify the 3'UTR of *Clcn3* (NM_173873). The 1st strand cDNA was prepared from total RNA prepared from mouse (C57BL/6j) spinal cord (SuperScript™ First-Strand Synthesis System for RT-PCR, Invitrogen) and PCR was conducted with 0.5 µl of cDNA and AccuPrime™ Pfx SuperMix (Invitrogen). Conditions are as follows: 95 deg 5min; 95 deg 15 sec, 58 deg 30 sec, 68 deg 2min40sec for 35

cycles; 68^0 deg for 10min . Amplicons are resolved on 1% agarose gel in TAE buffer, and an expected single band of the size above 2.5 kb was observed. Amplified DNA was purified (QIAquick PCR Purification Kit, QIAGEN), ligated into the cloning vector (Zero Blunt® TOPO® PCR Cloning Kit, Invitrogen), and further used to transform chemical competent cells (One Shot® TOP10 E. coli, Invitrogen). Colonies were picked randomly and inoculated into 5ml LB medium for miniprep. Clones were sequenced for verifying the identity (GATC Biotech, Konstanz). Correct recombinant vector was double digested with restriction enzymes EcoR V and Sac I to release the sequence of interest, and the released DNA was further collected by gel purification. In the meantime, the empty luciferase reporting vector (pmirGLO Dual-Luciferase miRNA Target Expression Vector, Promega) was treated with pme I and Sac I enzymes to generate appropriate ends for directional cloning. Ligation reaction was prepared with *clcn3 3'UTR* and the linearized reporter vector, and the reaction product was used to transform chemical competent cells as previously described. Positive clone was verified via restriction digestion with Xho I.

HEK cell culture and luciferase assay: HEK cells were cultured in Dulbecco's modified Eagle's medium (Gibco, 21969) containing 10% inactive FBS (Gibco, 10270), 200units/mL of Penicillin and 200µg/mL of Streptomycin (Gibco Pen-Strep, 15140) and 1X L-Glutamine (Gibco, 25030). Approximately 2.5×10^4 cells were plated into each well of 96 wells plate one day before the transfection and cultured in antibiotic free medium. Cells were then co-transfected with 600, 400 and 200 ng of either miR-1a-3p inhibitor or miR-1a-3p mirdian mimic together with 600 ng of *Clcn3* reporter vector into each well of 96 well plate using Dharmafect-1 transfection reagent (0.2µl/ well, Thermofischer Scientific, T-2001-02) following manufacturer's instructions. Forty-eight hours later, luciferase activities were quantified using Luc-Pair miR Luciferase Assay kit (Genecopoeia, LPFR-M100) and normalized to the respective control experiment.

Prediction of miRNA-1a-3p targets: miRNAs and their putative targets are collected from the fourteen different resources, namely Microcosm (Griffiths-Jones et al, 2008), TargetScan (Friedman et al, 2009), PicTar 7, Pictar13 (Krek et al, 2005), microRNA.org (Betel et al, 2008), miRDB (Wang & El Naqa, 2008), miRGen (Megraw et al, 2007), miRNAMap2 (Hsu et al, 2008), miRTarBase, miRTarBase WR (Hsu et al, 2011), PITA Top, PITA all (URL: http://genie.weizmann.ac.il/pubs/mir07/mir07_data.html), RepTar (Elefant et al, 2010), starBase (Yang et al, 2010), TarBase (Papadopoulos et al, 2009) were applied to identify the genes which represent putative targets of mmu-miR-1a-3p. miRTarBase_w and Tarbase databases

contain experimentally proven gene sets. miRTarBase_w and Tarbase databases contain experimentally proven gene sets. PITA-Top database represents top predictions having a full match 7- or 8-mer seed and a conservation score of 0.9 or higher where as PITA-all represents all predictions without any filter. In house generated Perl scripts were used to integrate and analyse the data obtained from above mentioned sources and extracted the genes which represent putative targets of mmu-miR-1a-3p.

Supplementary figures:

Suppl. Fig.1

Spinal cord cryo-sections

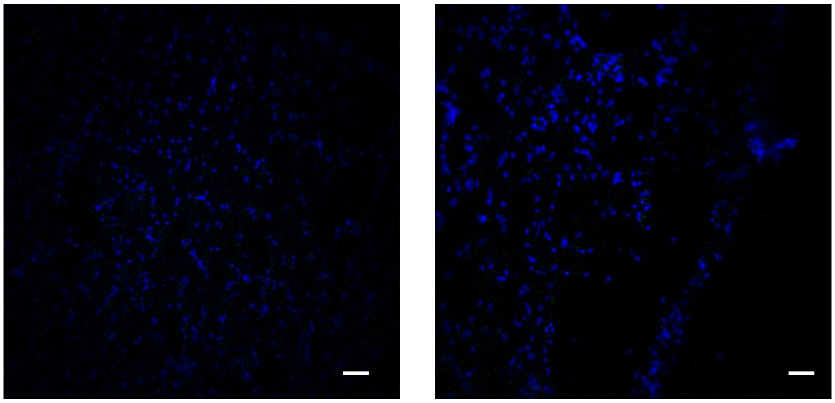

PBS-injected | **FAM-conjugated miRNA-inhibitor injected**

Suppl. Fig.1. Lack of uptake of intrathecally-administered miRNA-inhibitors in spinal cord neurons. Representative images from the cryo sections of lumbar spinal cord segment 4 are shown. Nucleus is stained for Dapi and imaged for Fluorescein amidite (FAM) signal with the same confocal imaging parameters & light intensity used for Fig.2 panel B. Scale bar represents 50 µm in both images.

Suppl. Fig. 2

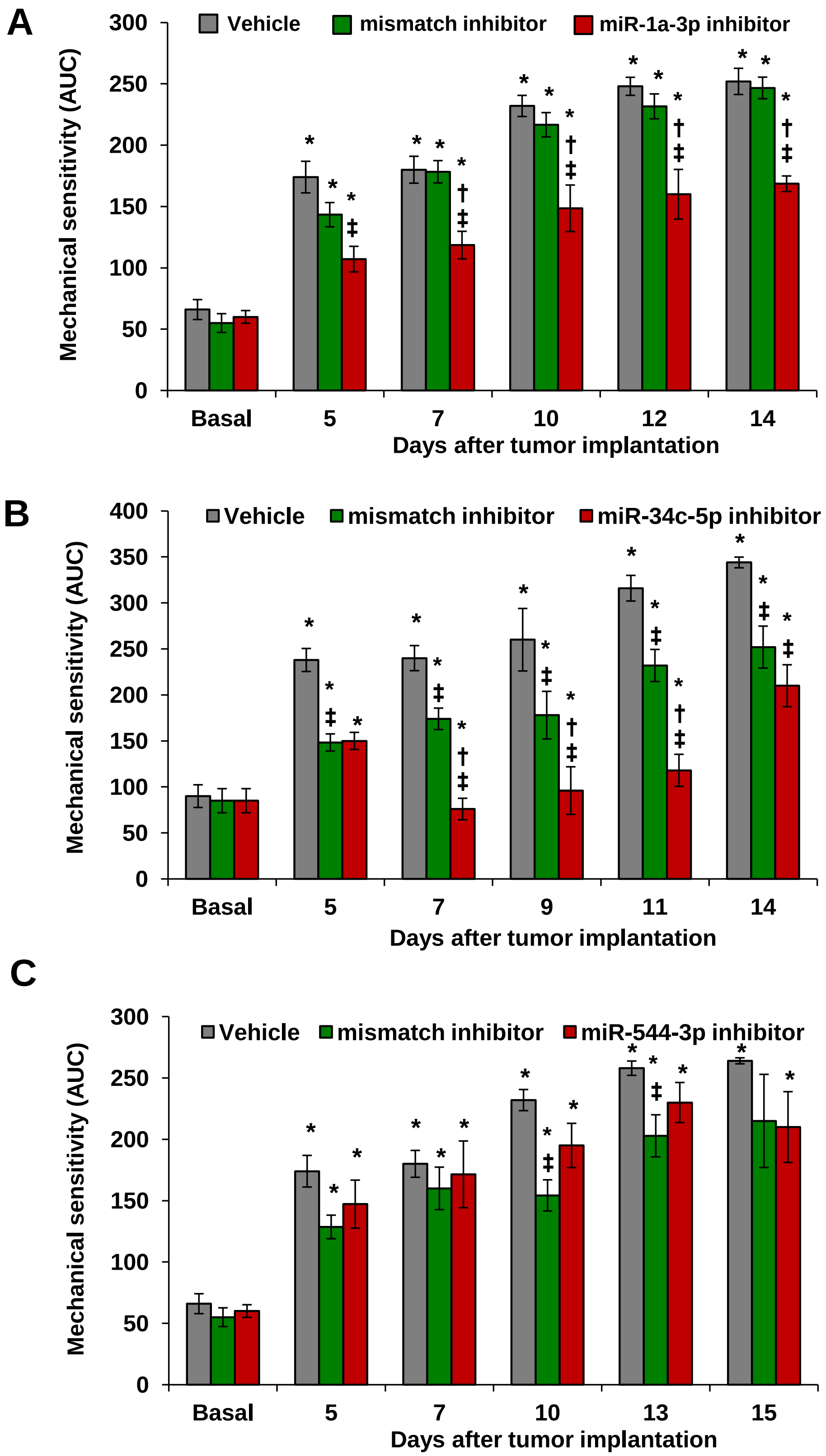
A
Vehicle
mismatch inhibitor
miR-1a-3p inhibitor
Mechanical sensitivity (AUC)
0
50
100
150
200
250
300
Basal
5
7
10
12
14
Days after tumor implantation
B
Vehicle
mismatch inhibitor
miR-34c-5p inhibitor
Mechanical sensitivity (AUC)
0
50
100
150
200
250
300
350
400
Basal
5
7
9
11
14
Days after tumor implantation
C
Vehicle
mismatch inhibitor
miR-544-3p inhibitor
Mechanical sensitivity (AUC)
0
50
100
150
200
250
300
Basal
5
7
10
13
15
Days after tumor implantation

Suppl. Fig.2. Functional validation of miRNAs upregulated in DRGs in tumor-bearing mice with respect to tumor-induced mechanical hypersensitivity. An integral of responsivity to mechanical stimuli over all von Frey forces tested (0.02 g to 1.0 g) in tumor-bearing mice represented as area under the curve (AUC). The experimental scheme employed is the same as described in Fig. 2A. In all panels * denotes P < 0.05 as compared to basal, In panel B & C † and ‡ denotes P ≤ 0.05 as compared to corresponding data point in the mismatch inhibitor or vehicle group respectively, two-way ANOVA of repeated measures followed by Bonferroni's multiple comparisons post-hoc test, n = at least 6 mice per group. In panels A, B & C, * in the vehicle group denotes P < 0.0001 from PID-5 through 15. In panel A * denotes P < 0.0001 for vehicle, mismatch inhibitor and miR-1a-3p-inhibitor groups from PID-5 through 14, † denotes P = 0.0031 on PID-7, 0.0002 on PID-10 and < 0.0001 on PID-12 & 14 and ‡ denotes P = 0.0003 on PID-5, 0.0016 on PID-7 and < 0.0001 on PID-10, 12 & 14; In panel B, * denotes P < 0.0001 for vehicle and mismatch inhibitor groups from PID-5 through 14, and 0.0115 on PID-11, < 0.0001 on PID-14 for the miR-34c-5p group, † denotes P < 0.0001 on PID-7, 9 & 11 and ‡ denotes P < 0.0001 on PID-5, 7, 9, 11 & 14 in the miR-34c-5p group and < 0.0001 on PID-5 & 14, 0.011 on PID-7, 0.0027 on PID-9 and 0.0001 on PID-11 in the mismatch inhibitor group; In panel C, * denotes P < 0.0001 on PID-7, 13 & 15 and 0.0002 on PID-10 for the mismatch inhibitor group; P = 0.0021 on PID-5 and < 0.0001 on PID-7 through 15 in the miR-544-3p-inhibitor group.

Suppl. Fig. 3

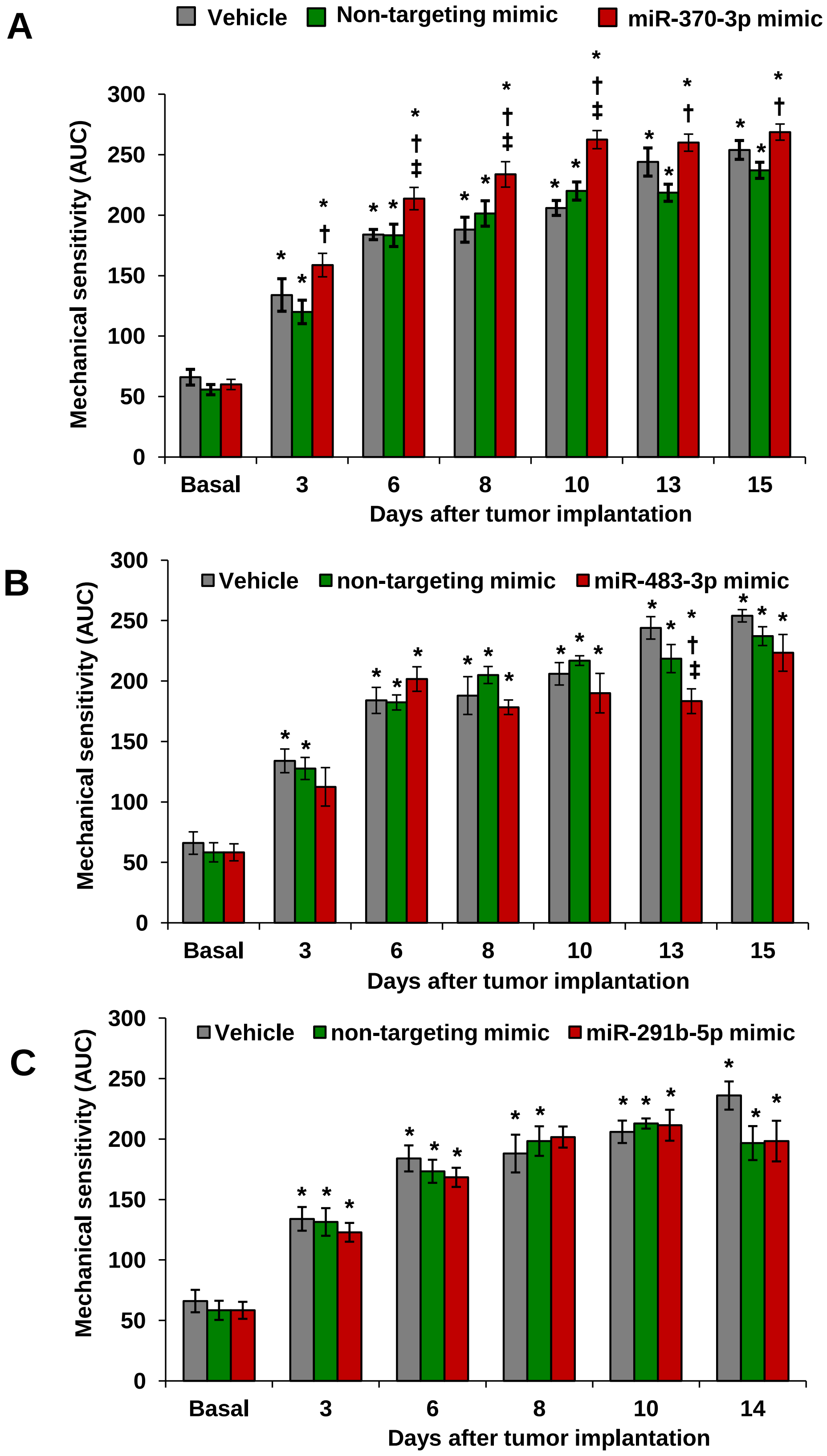
A
Vehicle
Non-targeting mimic
miR-370-3p mimic
Mechanical sensitivity (AUC)
0
50
100
150
200
250
300
Basal
3
6
8
10
13
15
Days after tumor implantation
B
Vehicle
non-targeting mimic
miR-483-3p mimic
Mechanical sensitivity (AUC)
0
50
100
150
200
250
300
Basal
3
6
8
10
13
15
Days after tumor implantation
C
Vehicle
non-targeting mimic
miR-291b-5p mimic
Mechanical sensitivity (AUC)
0
50
100
150
200
250
300
Basal
3
6
8
10
14
Days after tumor implantation

Suppl. Fig.3. Functional validation of miRNAs downregulated in DRGs in tumor-bearing mice with respect to tumor-induced mechanical hypersensitivity. An integral of responsivity to mechanical stimuli over all von Frey forces tested (0.02 g to 1.0 g) in tumor-bearing mice represented as area under the curve (AUC). The experimental scheme employed is the same as described in Fig. 2A. In all panels * denotes P ≤ 0.05 as compared to basal and in panels A & B † and ‡ denotes P ≤ 0.05 as compared to corresponding data point in the non-targeting mimic or vehicle group respectively, two-way ANOVA of repeated measures followed by Bonferroni's multiple comparisons post-hoc test, n = at least 6 mice per group. In panel A, B & C * denotes P < 0.0001 for all three groups; In panel A, † denotes P = 0.0124 on PID-3, 0.0353 on PID-6, 0.0178 on PID-8, 0.0058 on PID-10 & 13 and 0.0353 on PID-15. ‡ denotes P = 0.0353 on PID-6, 0.0026 on PID-8, 0.0001 and on PID-10. In panel B, † & ‡ denotes P = 0.0306 and 0.0002 respectively.

Suppl. Fig. 4

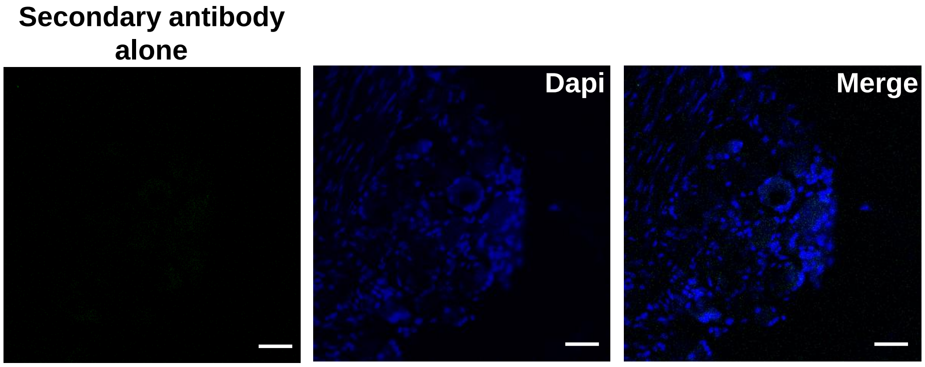

Suppl. Fig.4. Representative images from the cryosections of lumbar DRG to confirm the lack of autofluorescence or unspecific staining for images shown in Fig 5 panel E. Alexa-488 conjugated anti rabbit secondary antibody was incubated in the absence of anti-Clcn3 antibody. Nuclei were visualized with Dapi. Scale bar equals to 50 µm.

Suppl. Fig. 5

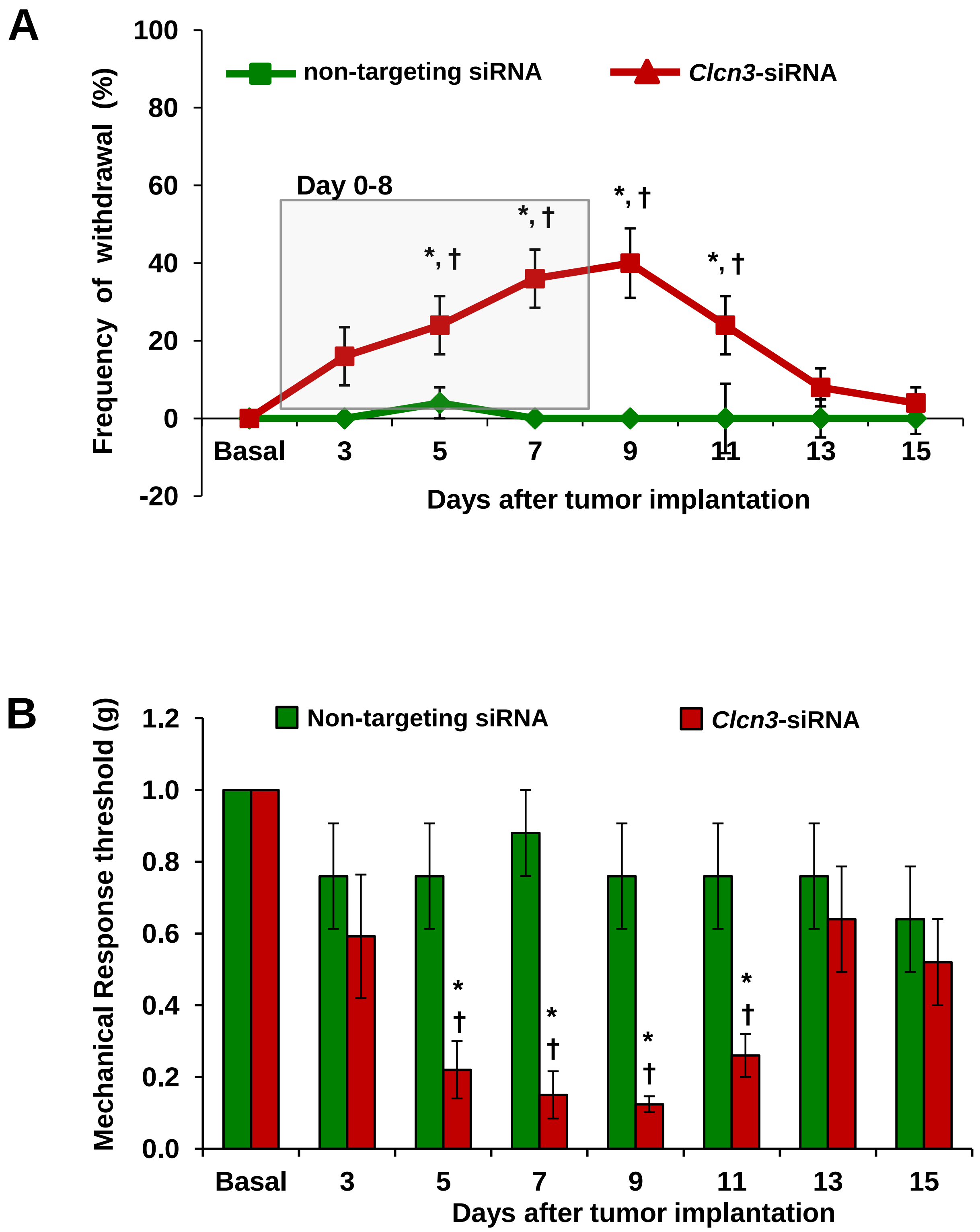
A
100
80
60
40
20
0
-20
Frequency of withdrawal (%)
non-targeting siRNA
Clcn3-siRNA
Day 0-8
*, †
Basal
3
5
7
9
11
13
15
Days after tumor implantation
B
1.2
1.0
0.8
0.6
0.4
0.2
0.0
Mechanical Response threshold (g)
Non-targeting siRNA
Clcn3-siRNA
*
†
Basal
3
5
7
9
11
13
15
Days after tumor implantation

Suppl. Fig.5. Effect of siRNA-mediated *Clcn3* knock-down in the DRGs on the basal mechanical sensitivity. (A) Change in frequency of paw withdrawal to plantar application of von Frey filament force of 0.02 g following intrathecal delivery of siRNA directed against *Clcn3* (red symbols) or non-targeting siRNA (green symbols) (B) magnitude of mechanical response threshold to von Frey filament strengths. In panels A & B, * denotes $P \leq 0.05$ as compared to basal and † denotes $P \leq 0.05$ as compared to corresponding non-targeting siRNA group; two-way ANOVA of repeated measures followed by Bonferroni's multiple comparisons post-hoc test, n = 6 mice per group. In panel A, * denotes P = 0.0038 on PID-5 & 11 and < 0.0001 on PID-7 & 9 and † denotes P = 0.0324 on PID-5, < 0.0001 on PID-7 & 9 and 0.0053 on PID-11. In panel B, * denotes P = 0.0003 on PID-5, 0.0038 on PID-7, < 0.0001 on PID-9, 0.0007 and PID-11, † denotes P = 0.0157 on PID-5, 0.0141 on PID-7, 0.0026 on PID-9, and 0.0295 on PID-11.

Supplementary Tables

Supplementary Table 1: Complete data over miRNAs which were found to be significantly upregulated or downregulated via microarray analysis in ipsilateral lumbar DRG on day 8 following induction of tumor growth in the calcaneous bone of the heel in mice as compared to sham surgery. Fold-change represents mean change in the mean expression of each miRNA in DRGs of tumor-bearing mice over mean expression from control DRGs (n=3 per group). Negative signs for fold-change indicate downregulated miRNAs. Refer to the methods section for statistical analyses.

miRNA ID-Ver 12	ID-miRBase Version 19	Accession number	mature sequence	Fold-change PID-4	Fold-change PID-8
mmu-miR-544	mmu-miR-544-3p	MIMAT0004941	AUUCUGCAUUUUUAGCAAGCUC	1.04	5.0
mmu-miR-200a	mmu-miR-200a-3p	MIMAT0000519	UAACACUGUCUGGUAACGAUGU	-1.02	4.6
mmu-miR-34b-5p	mmu-miR-34b-5p	MIMAT0000382	AGGCAGUGUAAUUAGCUGAUUGU	-1.02	4.5
mmu-miR-133a*	mmu-miR-133a-5p	MIMAT0003473	GCUGGUAAAAUGGAACCAAAU	1.03	4.0
solexa-5067-90	solexa-5067-90	NA	NA	-1.03	3.9
mmu-miR-154*	mmu-miR-154-3p	MIMAT0004537	AAUCAUACACGGUUGACCUAUU	1.05	3.6
mmu-miR-377	mmu-miR-377-3p	MIMAT0000741	AUCACACAAAGGCAACUUUUGU	1.14	3.5
mmu-miR-141	mmu-miR-141-3p	MIMAT0000153	UAACACUGUCUGGUAAAGAUGG	1.04	3.4
mmu-miR-376c	mmu-miR-376c-3p	MIMAT0003183	AACAUAGAGGAAAUUUCACGU	-1.09	3.4
mmu-miR-669j	mmu-miR-669j	MIMAT0005838	UGCAUAUACUCACAUGCAAACA	-1.04	3.2
solexa-2012-235	solexa-2012-235	NA	NA	1.05	3.2
mmu-miR-380-5p	mmu-miR-380-5p	MIMAT0000744	AUGGUUGACCAUAGAACAUGCG	-1.11	3.1
mmu-miR-142-5p	mmu-miR-142-5p	MIMAT0000154	CAUAAAGUAGAAAGCACUACU	1.18	3.1
mmu-miR-34c	mmu-miR-34c-5p	MIMAT0000381	AGGCAGUGUAGUUAGCUGAUUGC	-1.27	3.0
mmu-miR-181a-1*	mmu-miR-181a-1-3p	MIMAT0000660	ACCAUCGACCGUUGAUUGUACC	-1.07	2.9
mmu-miR-582-3p	mmu-miR-582-3p	MIMAT0005292	UAACCUGUUGAACAACUGAAC	-1.09	2.9

mmu-miR-369-3p	mmu-miR-369-3p	MIMAT0003186	AAUAAUACAUGG UUGAUCUUU	-1.04	2.9
solexa-4179-110	solexa-4179-110	NA	NA	1.07	2.9
mmu-miR-380-3p	mmu-miR-380-3p	MIMAT0000745	UAUGUAGUAUGG UCCACAUCUU	1.03	2.8
mmu-miR-1	mmu-miR-1a-3p	MIMAT0000123	UGGAAUGUAAAG AAGUAUGUAU	-1.30	2.8
mmu-miR-496	mmu-miR-496a-3p	MIMAT0003738	UGAGUAUUACAU GGCCAAUCUC	-1.17	2.7
mmu-miR-130b	mmu-miR-130b-3p	MIMAT0000387	CAGUGCAAUGAU GAAAGGGCAU	1.01	2.7
mmu-miR-499	mmu-miR-499-5p	MIMAT0003482	UUAAGACUUGCA GUGAUGUUU	-1.06	2.7
mmu-miR-16*	mmu-miR-16-1-3p	MIMAT0004625	CCAGUAUUGACU GUGCUGCUGA	1.31	2.7
mmu-miR-15b*	mmu-miR-15b-3p	MIMAT0004521	CGAAUCAUUAUU UGCUGCUCUA	1.04	2.6
mmu-miR-376c*	mmu-miR-376c-5p	MIMAT0005295	GUGGAUAUUCCU UCUAUGUUUA	1.01	2.6
mmu-miR-483*	mmu-miR-483-3p	MIMAT0003120	UCACUCCUCCCC UCCCGUCUU	-1.02	-5.3
solexa-5306-86	solexa-5306-86	NA	NA	1.02	-5.1
mmu-miR-423-5p	mmu-miR-423-5p	MIMAT0004825	UGAGGGGCAGA GAGCGAGACUUU	-1.08	-4.8
mmu-miR-1224	mmu-miR-1224-5p	MIMAT0005460	GUGAGGACUGG GGAGGUGGAG	1.15	-4.2
mmu-miR-323-5p	mmu-miR-323-5p	MIMAT0004638	AGGUGGUCCGU GGCGCGUUCGC	-1.04	-4.1
mmu-miR-760	mmu-miR-760-3p	MIMAT0003898	CGGCUCUGGGU CUGUGGGGA	-1.03	-4.0
mmu-miR-466i	mmu-miR-466i-3p	MIMAT0005834	AUACACACACAC AUACACACUA	-1.18	-3.9
mmu-miR-681	mmu-miR-681	MIMAT0003458	CAGCCUCGCUGG CAGGCAGCU	-1.07	-3.8
mmu-miR-1188	mmu-miR-1188-5p	MIMAT0005843	UGGUGUGAGGU UGGGCCAGGA	1.11	-3.6
solexa-403-1161	solexa-403-1161	NA	NA	-1.24	-3.6
mmu-miR-291b-5p	mmu-miR-291b-5p	MIMAT0003189	GAUCAAAGUGGA GGCCCUCUCC	1.10	-3.4
solexa-1278-371	solexa-1278-371	NA	NA	1.05	-3.4
mmu-miR-702	mmu-miR-702-3p	MIMAT0003492	UGCCCACCCUUU ACCCCGCUCC	1.16	-3.3
mmu-miR-370	mmu-miR-370-3p	MIMAT0001095	GCCUGCUGGGG UGGAACCUGGU	1.41	-3.2
mmu-miR-466f-3p	mmu-miR-466f-3p	MIMAT0004882	CAUACACACACA CAUACACAC	-1.09	-3.2
mmu-miR-877*	mmu-miR-877-3p	MIMAT0004862	UGUCCUCUUCUC CCUCCUCCCA	-1.27	-3.1
mmu-miR-669c	mmu-miR-669c-5p	MIMAT0003479	AUAGUUGUGUGU GGAUGUGUGU	1.00	-3.1

mmu-miR-877	mmu-miR-877-5p	MIMAT0004861	GUAGAGGAGAUG GCGCAGGG	1.04	-3.1
mmu-miR-466g	mmu-miR-466g	MIMAT0004883	AUACAGACACAU GCACACACA	-1.22	-3.0
mmu-miR-874	mmu-miR-874-3p	MIMAT0004853	CUGCCCUGGCCC GAGGGACCGA	1.03	-3.0
mmu-miR-673-5p	mmu-miR-673-5p	MIMAT0003739	CUCACAGCUCUG GUCCUUGGAG	-1.04	-2.9
mmu-miR-298	mmu-miR-298-5p	MIMAT0000376	GGCAGAGGAGG GCUGUUCUUCCC	1.12	-2.9
mmu-miR-466d-3p	mmu-miR-466d-3p	MIMAT0004931	UAUACAUACACG CACACAUAG	-1.27	-2.9
mmu-miR-714	mmu-miR-714	MIMAT0003505	CGACGAGGGCC GGUCGGUCGC	-1.08	-2.9
mmu-miR-197	dead entry	NA	NA	-1.11	-2.8
mmu-miR-296-5p	mmu-miR-296-5p	MIMAT0000374	AGGGCCCCCCCU CAAUCCUGU	1.24	-2.8
mmu-miR-1198	mmu-miR-1198-5p	MIMAT0005859	UAUGUGUUCCUG GCUGGCUUGG	1.10	-2.7
mmu-miR-31	mmu-miR-31-5p	MIMAT0000538	AGGCAAGAUGCU GGCAUAGCUG	1.01	-2.6
mmu-miR-218-1*	mmu-miR-218-1-3p	MIMAT0004665	AAACAUGGUUCC GUCAAGCACC	-1.03	-2.6
mmu-miR-483	mmu-miR-483-5p	MIMAT0004782	AAGACGGGAGAA GAGAAGGGAG	-1.02	-2.6
mmu-miR-467a*,d*	mmu-miR-467a-3p	MIMAT0002108	CAUAUACAUACA CACACCUACA	-1.27	-2.5

Supplementary Table 2: Sequences of LNA-based miRNA-inhibitors which were custom designed for miRNA inhibition in the DRGs *in vivo*. Highlighted nucleotides represent introduced mis-matches as compared to the original sequences.

miRNA-name	Inhibitor sequence (5'-3')	Mismatch control inhibitor sequence (5'-3')
mmu-miR-1a-3P	FAM- TCTTTACATTCC	FAM- TCATTTCTATCC
mmu-miR-34c-5P	FAM-CTAACTACACTGCC	FAM- CTATCAACTCAGCC
mmu-miR-544-3P	FAM-GAGCTTGCTAAAAATGCAGA	FAM- GAGCTTACAATAGATGCAGA

Supplementary Table 3: Summary of top 10 genes which represent predictions as targets of miR-1a-3p via *in silico* analysis using 14 different algorithms. *(V) represents previously validated targets. 'Score' in the last column represents number of algorithms which predict a corresponding gene as a putative target.

**Name**	**MGI designation**	**Microcosm**	**TargetScan**	**Pictar7**	**Pictar13**	**MiRNA.org**	**Mirdb**	**Mirgen**	**Mirnamap**	**MirTarBase**	**MirTarBase_w(V)***	**Pita_top**	**Pita_all**	**Reptar**	**Starbase**	**Tarbase(V)***	**Score**
Gja1	Gap junction protein, alpha 1	✓				✓		✓	✓	✓	✓	✓	✓	✓	✓		10
Hspd1	Heat shock protein 1 (chaperonin)	✓				✓		✓	✓	✓	✓	✓	✓	✓			9
Igf1	Insulin-like growth factor 1	✓				✓			✓	✓	✓	✓	✓	✓			8
Anxa2	Annexin A2	✓				✓		✓	✓			✓	✓	✓	✓		8
Clcn3	Chloride channel 3	✓				✓		✓	✓			✓	✓	✓	✓		8
Mab21l1	Mab-21-like 1 (C. elegans)	✓				✓		✓	✓			✓	✓	✓	✓		8
Trim2	Tripartite motif-containing 2	✓				✓		✓	✓			✓	✓	✓			7
Hand2	Heart and neural crest derivatives expressed transcript 2					✓		✓		✓	✓		✓	✓			6
Fn1	Fibronectin 1	✓				✓		✓		✓	✓			✓			6
Cdk9	Cyclin-dependent kinase 9 (CDC2-related kinase)	✓				✓		✓		✓	✓		✓				6

References:

Betel D, Wilson M, Gabow A, Marks DS, Sander C (2008) The microRNA.org resource: targets and expression. *Nucleic Acids Res* **36**(Database issue)**:** D149-153

Elefant N, Berger A, Shein H, Hofree M, Margalit H, Altuvia Y (2010) RepTar: a database of predicted cellular targets of host and viral miRNAs. *Nucleic Acids Res* **39**(Database issue)**:** D188-194

Friedman RC, Farh KK, Burge CB, Bartel DP (2009) Most mammalian mRNAs are conserved targets of microRNAs. *Genome Res* **19**(1)**:** 92-105

Griffiths-Jones S, Saini HK, van Dongen S, Enright AJ (2008) miRBase: tools for microRNA genomics. *Nucleic Acids Res* **36**(Database issue)**:** D154-158

Hsu SD, Chu CH, Tsou AP, Chen SJ, Chen HC, Hsu PW, Wong YH, Chen YH, Chen GH, Huang HD (2008) miRNAMap 2.0: genomic maps of microRNAs in metazoan genomes. *Nucleic Acids Res* **36**(Database issue)**:** D165-169

Hsu SD, Lin FM, Wu WY, Liang C, Huang WC, Chan WL, Tsai WT, Chen GZ, Lee CJ, Chiu CM, Chien CH, Wu MC, Huang CY, Tsou AP, Huang HD (2011) miRTarBase: a database curates experimentally validated microRNA-target interactions. *Nucleic Acids Res* **39**(Database issue)**:** D163-169

Krek A, Grun D, Poy MN, Wolf R, Rosenberg L, Epstein EJ, MacMenamin P, da Piedade I, Gunsalus KC, Stoffel M, Rajewsky N (2005) Combinatorial microRNA target predictions. *Nat Genet* **37**(5)**:** 495-500

Megraw M, Sethupathy P, Corda B, Hatzigeorgiou AG (2007) miRGen: a database for the study of animal microRNA genomic organization and function. *Nucleic Acids Res* **35**(Database issue)**:** D149-155

Papadopoulos GL, Reczko M, Simossis VA, Sethupathy P, Hatzigeorgiou AG (2009) The database of experimentally supported targets: a functional update of TarBase. *Nucleic Acids Res* **37**(Database issue)**:** D155-158

Schweizerhof M, Stosser S, Kurejova M, Njoo C, Gangadharan V, Agarwal N, Schmelz M, Bali KK, Michalski CW, Brugger S, Dickenson A, Simone DA, Kuner R (2009) Hematopoietic colony-stimulating factors mediate tumor-nerve interactions and bone cancer pain. *Nat Med* **15**(7)**:** 802-807

Wang X, El Naqa IM (2008) Prediction of both conserved and nonconserved microRNA targets in animals. *Bioinformatics* **24**(3)**:** 325-332

Yang JH, Li JH, Shao P, Zhou H, Chen YQ, Qu LH (2010) starBase: a database for exploring microRNA-mRNA interaction maps from Argonaute CLIP-Seq and Degradome-Seq data. *Nucleic Acids Res* **39**(Database issue)**:** D202-209
